# Resources modify the associations between kin and fertility in historical Finland

**DOI:** 10.1017/ehs.2026.10060

**Published:** 2026-07-03

**Authors:** Milla Salonen, Mirkka Lahdenperä, Takayuki Hiraoka, Jari Saramäki, Virpi Lummaa

**Affiliations:** 1 Department of Biology, University of Turku, Finland; 2 Department of Computer Science, Aalto University, Finland

**Keywords:** inclusive fitness, kin competition, fertility, marriage, socioeconomic status

## Abstract

Inclusive fitness theory predicts that to maximize fitness, individuals should invest in kin, but relatives can also compete for resources. These opposing forces can have opposite effects on fertility, which was further constrained by marital and inheritance norms in traditional Western societies. How available resources influence this balance between helping and competing remains poorly understood. To investigate whether kin help or harm fertility, we used genealogical records from a population registry to study how relatives alive at age 15 were associated with marriage and lifetime number of children in Finnish people born 1752–1879, comparing landowning and landless individuals. Our results show that for focals born to landowning families, both older and younger siblings were associated with a higher age at first marriage, but the maternal grandmother and younger sisters were positively associated with fertility. These associations also differed by the focal’s sex. Among the landless, siblings were also relatively weakly associated with poor marital outcomes, but not with the lifetime number of children. Relatives were not associated with lifetime childlessness. These results suggest that a high level of resources may intensify both positive and negative associations between kin and fertility, possibly through inheritance patterns, competition for resources and childcare help.

## Graphical abstract

**Figure:**
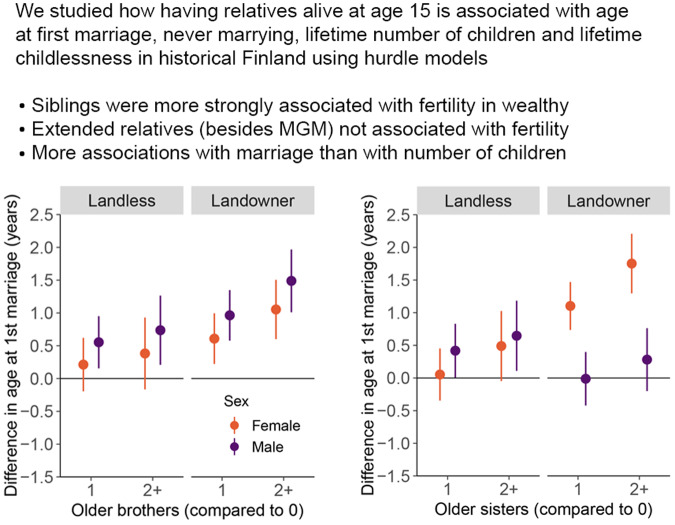

## Social media summary

In 18th- and 19th-century Finland, having living siblings was linked to marriage and childbearing – not always positively.

## 1. Introduction

An individual’s total fitness is the combination of their personal fitness and the fitness of their genetic relatives (Hamilton, 1964). This inclusive fitness theory has been used to explain, for example, cooperative breeding, meaning allomaternal help in rearing offspring (Hrdy, 2007). Alloparental help has been suggested to be essential in past human populations (Hrdy, 2007). The more closely related the helper is, the greater indirect fitness benefits they can gain from helping (Hamilton, 1964). The most common helpers in human societies are pre-reproductive siblings, often called helpers-at-the-nest, and post-reproductive grandmothers, who both gain fitness benefits from helping as they do not reproduce themselves (Emlen, 1994; Sear & Coall, 2011; Sear & Mace, 2008). Due to paternity uncertainty, relatives on the maternal side are expected to invest more in helping (Queller, 1997). Even unrelated individuals, especially pre-reproductive individuals, can sometimes benefit from helping, as the experience of caretaking may improve the survival of their own future offspring (Hrdy, 2007). However, individuals will likely maximize their own fitness even to the detriment of relatives (Hamilton, 1964).

As inclusive fitness theory predicts, some studies have discovered that the presence of kin increases fertility (realized reproduction) in humans (Mathews & Sear, 2013; Sear & Coall, 2011), although some show contrasting results. For example, some studies have discovered that the fertility of couples is positively influenced by their parents (Chapman et al., 2021; Dillon et al., 2024; Okun & Stecklov, 2021; Sear, 2018; Sear et al., 2003; Snopkowski & Sear, 2013; Tymicki, 2004, 2008). The presence of parents-in-law has similarly been associated with higher fertility in women (Dillon et al., 2024; Hacker et al., 2021; Sear & Coall, 2011; Sear et al., 2003; Willführ et al., 2022), whereas a woman’s own parents may decrease fertility due to concerns about maternal well-being (Hacker et al., 2021; Sear & Coall, 2011). In contrast, siblings have been previously mostly associated with lower fertility (Borgerhoff Mulder, 1998; Gibson & Gurmu, 2011; Low, 1991; Mace, 1996; Yang & Spencer, 2022, but see Rotering & Bras, 2015).

Relatives can influence fertility through several different mechanisms. Relatives may provide fitness benefits through help with childcare (Sear, 2018), which can reduce the stress related to childcare and shorten inter-birth intervals (Chapman et al., 2021; Chapman & Lummaa, 2024; Dillon et al., 2024, 2024; Lahdenperä et al., 2004; Szabó et al., 2017). They may also increase fertility through peer pressure, shared knowledge, and transmission of attitudes towards childbearing (Newson et al., 2005; Sear, 2018). For example, grandparents’ desire for grandchildren can exert social pressure on their children’s reproductive decisions (Axinn et al., 1994; Newson et al., 2005). Relatives can also indirectly increase fertility through harmful effects on child survival, as the death of a child is strongly associated with a shorter time to transition to a subsequent birth (Chapman et al., 2021; Sear et al., 2003; Tymicki, 2008). In contrast, having relatives present can decrease fertility because they compete for the same resources necessary for childbearing and childcare (Sear, 2018; Van Dyken, 2010).

Relatives can also influence fertility indirectly through marriage. In humans, fertility is constrained not only by fecundity (potential reproductive capacity) but also by societal norms. In many historical Western countries, fertility was almost fully constrained by marriage, as extramarital relations were strongly disapproved of (Newson et al., 2005; Sundin, 1992). However, entering marriage required adequate resources to set up a household (Newson et al., 2005), and the minimum age of spouses was also regulated in some countries (Moring, 2003). Like fertility, kin can influence marriage propensity in two possible directions. On the one hand, kin may help find potential spouses, arrange marriages, and provide social pressure towards marrying, increasing marriage propensity and thus enabling future fertility. On the other hand, kin, such as siblings (Pettay et al., 2020), may compete for spouses and for the resources required to marry. For example, in some societies, the oldest son gained the largest inheritance and was expected to marry before other siblings (Moring, 1998), likely constraining the marriage opportunities of his siblings. These effects of kin on marriage may be similar to or different from their effects on fertility (number of children), and not much is known about the influence of both close and more distant kin on marriage.

The extent of available resources can modify the association between kin and fertility outcomes. Altruism towards kin may be diminished if individuals have to compete for limited resources within their local population (Boyd, 1982; Frank, 1998; Hamilton, 1964). Therefore, abundant resources may favour cooperation, whereas scarcity could intensify competition. This effect has been studied in, for example, bacteria and insects (Brockhurst et al., 2008; Connelly et al., 2017; West et al., 2001). Some studies on human populations have indicated that, in line with the resource availability theory, kin are more beneficial for fertility among the wealthy. One study discovered that low socioeconomic status (SES) exaggerated the harmful effect of long co-residence with parents on fertility outcomes (Schaffnit & Sear, 2014). In contrast, when resources are transferred intergenerationally through, for example, inheritance, siblings of wealthier families can compete for resources (Gibson & Gurmu, 2011; Mace, 1996; Moring, 1998), whereas children born to poor families may be less affected by sibling competition, as they may receive no inheritance regardless of the number of siblings (Bengtsson et al., 2014; Moring, 1998, 2003). Furthermore, childcare help may be especially important in low-income and historical societies, as caring for children may physiologically limit future childbearing ability. Supporting this, mothers have been shown to increase the fertility of their daughters in low-income or historical societies (Chapman et al., 2021; Sear, 2018), whereas results are mixed in high-income societies, possibly because women can utilize other means of support, such as formal childcare (Sear, 2018). Similarly, one study found that mother’s presence increased fertility among the landless, but decreased it among farmers (Johow & Voland, 2012). While previous studies have shown that associations of more distant kin, such as aunts and uncles, with child survival can vary by SES (Borgerhoff Mulder, 2007; Hadley, 2004), the relationship between distant kin and fertility outcomes across SES groups remains unexplored. In sum, resources can modify the link between kin and fertility in two opposite directions: kin may be more beneficial for fertility among the wealthy if cooperation increases with resource abundance, but more harmful if the wealthy compete more strongly for intergenerationally transferred resources.

The historical Finnish society was patrilocal, with paternal kin commonly residing nearby (Moring, 1999). Furthermore, residing in the same household with paternal grandparents was common (Moring, 2003). In Eastern Finland, households comprising multiple brothers’ joint families were common, and these households were usually larger due to the labour force needed to practice the common slash-and-burn cultivation (Moring, 1999). Among landowners, the oldest son inherited the family farm, but was also required to care for his elderly parents and younger siblings (Moring, 1998, 1999). Importantly, the oldest son was expected to marry first, and only after his marriage could younger siblings do the same (Moring, 1999, 2003). Marrying also required resources, as husbands were obliged to provide for their families, and the bride’s family was expected to provide a dowry (Moring, 1998). Extramarital relations and divorce were forbidden (Sundin, 1992), and therefore, it is unlikely that there are extramarital children unaccounted for (Larmuseau et al., 2016, 2019). Historical Finland was a poor agrarian society (Soininen, 1974), with farming as the primary livelihood, but in the coastal areas of Southwest Finland, fishing improved food predictability (Lummaa et al., 1998). Social status in historical Finland was defined by wealth and estate. The wealthiest were members of higher estates, nobility and clergy, who composed a very small proportion of the population (Uotila, 2022; Voutilainen, 2017). For most of society, status was defined by landownership: those with most status were the freeholding peasants, who owned and cultivated their own land. Outside of estate were tenant farmers, who rented but did not own land, craftsmen, and labourers, as well as servants and the unemployed, who were the poorest members of the society (Uotila, 2022; Voutilainen, 2017).

Understanding the links between kin and fertility and their modifiers is important because they may have shaped the evolution of key human life–history traits, such as dispersal patterns, menopause, and reproductive strategies. In this study, we investigate the association of the presence of both close family members and more distant relatives at the focal’s age of 15 with the probability to marry, age at first marriage, lifetime childlessness, and the lifetime number of children in historical Finland, in people born between 1752 and 1879. Age 15 was the minimum legal age for marrying (Moring, 2003), and it can be assumed that relatives alive at this life stage could influence these fertility outcomes. As reproductive events may occur even decades later, the number of kin alive at age 15 reflects the early life social environment. Therefore, in this study, we assess the long-term associations of having kin alive during adolescence. We chose the lifetime number of children, instead of yearly reproduction, as the total number of children during the lifetime highlights a great investment in reproduction. We study the total number of births, rather than children surviving to a certain age, to separate associations of kin on fertility from associations of kin on child survival (Lahdenperä et al., 2025). We consider relatives with a genetic relatedness of at least 0.125 to the focal (as calculated from a pedigree), and include both vertical relatives (parents, grandparents, aunts, and uncles) and horizontal relatives (siblings and their children and cousins) from both paternal and maternal lineages. We use mixed-effects hurdle models and include three-way interactions between focal’s sex, SES, and relative type to assess whether kin–fertility associations differ between socioeconomic classes and sexes. In these models, we also control for birth region, birth cohort, and family identity (to control for maternal effects).

We formulated several testable hypotheses:

*Hypothesis 1*: Having close relatives alive at age 15 would be more strongly associated with fertility outcomes than having more distantly related relatives alive.

*Alternative hypothesis 1.1*: Close relatives would be associated with fertility positively (improve fertility) in line with kin selection theory (Hamilton, 1964).

*Alternative hypothesis 1.2*: Close relatives would be associated with fertility negatively (decrease fertility) due to competition for resources, such as inheritance.

*Hypothesis 2*: Having vertical relatives, especially parents, alive at age 15 would be more positively associated with fertility outcomes due to intergenerational transfers (parental and grandparental investment) compared to having horizontal kin (siblings and cousins) alive (Lee, 2008).

*Hypothesis 3*: Having maternal relatives alive at age 15 would be more positively associated with fertility outcomes than having paternal relatives alive, as previous studies in this population have discovered negative associations between paternal kin and other fitness outcomes (Chapman et al., 2019; Lahdenperä et al., 2025; Pettay et al., 2016).

*Hypothesis 4*: Having kin alive at age 15 would be more strongly associated with fertility outcomes in the wealthy than in the poor.

*Alternative hypothesis 4.1*: Kin would be more positively associated with fertility among the wealthy than among the poor, as those with many resources may cooperate more (Boyd, 1982; Frank, 1998).

*Alternative hypothesis 4.2*: Kin would be more negatively associated with fertility among the wealthy than among the poor, as competition for resources such as inheritance may be stronger among the wealthy.

*Hypothesis 5*: Having relatives alive at age 15 may have contrasting associations with different fertility outcomes. For example, having deceased parents may be associated with a lower age at first marriage due to gaining inheritance, but also with a lower lifetime number of children due to the absence of alloparents.

## 2. Materials and methods

### 2.1. Study population

We studied the probability of ever marrying and age at first marriage, and the lifetime number of children before the onset of the demographic transition in a longitudinal family lineage dataset. This dataset was derived from extensive, high-quality population records collected by the Lutheran Church of Finland. Collecting population records was mandatory by law since 1749 (Georg Luther, 1993), but the collection of these records had already started in the 17th century. These registers collected information on all births, marriages, migrations, occupations, and deaths. Historical demographic datasets are often biased by the disappearance of individuals (stayer bias). In historical Finland, however, both immigration to a parish and emigration out of a parish were recorded, and we particularly focused on tracking individuals until death. Our dataset contains family lineages that initially originated from eight parishes: Hiittinen, Kustavi, and Rymättylä (Southwest Finland region); Ikaalinen and Tyrvää (Pirkanmaa region); Pulkkila (Northern Ostrobothnia region); and Jaakkima and Rautu (Karelia region, annexed to Russia after the Second World War).

### 2.2. Cohort

#### Probability of ever marrying and age at first marriage

This study sample includes focals who were born between 1752 and 1879 and either married during their fertile years between the ages of 15 and 45 or did not marry and survived until 37.2 years of age, when 95% of ever-married people in our sample had married. We only retained unmarried focals whose full life history was known, and therefore we are likely to have complete information on their marital status and fertility. To be able to control for a possible family effect (Kolk, 2014), we only retained focals whose mother’s identity was known. We also excluded focals that had missing values in the explanatory and outcome variables listed in the section below. The final sample size was 13,856 focals from 5528 mothers. The data included 3197 men and 3324 women from landless families and 3471 men and 3864 women from landowning families. Of the landless, 95.1% of men and 92.2% of women married during their lifetime. Of the landowning, 93.5% of men and 93.3% of women married. The mean age at first marriage was 26.1 years for landless men, 25.1 for landless women, 26.4 for landowner men, and 24.4 for landowner women.

#### Lifetime childlessness and lifetime number of children

From the previously described study sample (born between 1752 and 1879, either married or survived unmarried until 37.2 years of age), we only included those focals from the sample who had married at least once, survived until the age of 45 years, and had their full life history known. The final sample sizes were 3514 (from 1800 mothers) for the association of maternal kin with the lifetime number of children and 3468 (from 1800 mothers) for the association of paternal kin with the lifetime number of children. A total of 1286 focals appear in both datasets, while 2228 focals were only included in the maternal kin dataset and 2182 focals were only included in the paternal kin dataset. In the combined dataset, 8.1% of landless men and 5.7% of landless women remained childless during their lifetime, whereas 7.2% of landowning men and 6.0% of landowning women remained childless. Among those focals who had at least one child, the mean number of children was 6.2 for landless men, 5.9 for landless women, 6.4 for landowning men, and 6.1 for landowning women.

### 2.3. Variables

In the hurdle model of age at first marriage, the outcome combined information about whether the focal was married during their lifetime and their age at first marriage in years (AFM). In this variable, focals who never married were assigned a value of 0, while for those who married, this variable recorded their age at first marriage in years. The age at first marriage was calculated as the difference between the precise date of the first marriage record and the date of birth. In the hurdle model of lifetime number of children, the number of children was treated as a discrete numerical variable.

For all focals, the birth parish was recorded. We classified these parishes into three larger regions: Southwest Finland (including, for example, parishes Hiittinen, Kustavi, and Rymättylä), Central Finland (including, for example, parishes Ikaalinen and Tyrvää), and Northern and Eastern Finland (including, for example, parishes Pulkkila, Jaakkima, and Rautu). This classification is based on genetic, cultural, and linguistic differences between the regions (Honkola et al., 2018, 2019; Kerminen et al., 2017).

The combined occupations of the focals’ parents were used to classify the focals into two social classes: landowning (e.g. landowners, but also noblemen, clergy, and merchants) and landless (e.g. tenant farmers, craftsmen, servants, and dependent lodgers). Classification was based on the father’s occupation, unless the father was unknown; in which case, the mother’s occupation was used. This classification has captured key differences in lifespan, marriage, and fertility (Liu et al., 2012; Pettay et al., 2007). We used parental social classes, as we examined the presence of kin at age 15 and marriage probability, and therefore, parental social class is likely to influence these outcomes.

We grouped birth year into 10-year birth cohorts (1750–1870, 13 cohorts) to account for possible non-linear cohort effects. For models of lifetime number of children, we also categorized age at first marriage to control for its association with the outcome: under 20 years of age, 20–25 years of age, 25–30 years of age, 30–35 years of age, and over 35 years of age.

We determined the number of 18 different relatives (including mother, father, siblings, nieces and nephews, grandparents, aunts, uncles, and cousins) with the minimum relatedness of 0.125. These relatives were divided by lineage (paternal and maternal) and sex (except cousins, for which numbers of female and male cousins were highly correlated), with siblings also split into older and younger siblings. The number of relatives was recorded when the focal was 15 years old, which was the legal minimum age of marriage for women (18 for men) during the study period (Moring, 2003). Most focals had either none or few of each relative and, thus, the numbers are right-skewed. Therefore, all relatives except individual parents and grandparents were coded as multilevel categorical variables (Supplementary Tables S13 and S14). Multilevel variables rather than simple presence/absence were used, as we assumed that a higher number of kin, such as sibling types, and not just their presence, would be associated with fertility outcomes.

### 2.4. Data analyses

#### Number of relatives and marriage

All analyses were conducted in R version 4.2.2 (R Core Team, 2022). We utilized mixed-effects hurdle models to examine the association of relatives with marriage using the package *glmmTMB* (Brooks et al., 2017; McGillycuddy et al., 2025). Hurdle models are two-part models that simultaneously but separately model a binomial component and a numerical component (Cragg, 1971; Mabire-Yon, 2025). Hurdle models assume that datapoints must cross a binary ‘hurdle’, after which their distribution in the numerical component is modelled (Cragg, 1971; Mabire-Yon, 2025). In our case, the hurdle to cross is getting married in one’s lifetime. If a person married in their lifetime, we modelled their age at first marriage. We used the combined marriage variable (0 for those who never married, age at first marriage for those who married) as the outcome. Before fitting models, we ensured adequate sample sizes in both the never married and married groups across all levels of categorical variables by cross-tabulating.

We first fit full models separately for paternal relatives, maternal relatives, and siblings (also including siblings’ children). These full models included all relatives of the lineage (for example, the paternal model included father, paternal grandfather, paternal grandmother, paternal aunts, paternal uncles, and paternal cousins) and focal’s sex (male/female), parental SES (landless/landowner), and birth region (three-level categorical) as fixed covariates and mother’s identity and birth cohort as random effects. For these full models, we examined model fit visually using the package *DHARMa* (Hartwig, 2022) and assessed multicollinearity with the variance inflation factor (VIF) using the package *car* (Fox & Weisberg, 2019). The models reached good model fit with the normal distribution (family = ‘gaussian’), with the zero-inflation formula including all explanatory variables and their interactions.

We then performed model selection to assess which relatives should be included in the final model using the package *MuMIn* (Bartoń, 2025). This model selection was performed separately for the binomial outcome (never married/did marry) and the continuous outcome (age at first marriage) due to computational restrictions. Covariates were required to be in all models. We selected the models with the lowest AICc (corrected Akaike information criterion) value and fit a hurdle model including all relative types retained in the best-fitting binomial and continuous models. To compare the probability to marry and age at first marriage within each sex and SES class, we also fit three-way interactions between focal’s sex, parental SES, and relatives. As neither paternal nor maternal kin were significantly associated with the outcome in this model, we fit a final model with only siblings to maximize the sample size. This final model included siblings that were selected in model selection and their three-way interaction with focal’s sex and parental SES, as well as region as a fixed covariate, and mother’s identity and birth cohort as random effects. This model, therefore, controls for regional effects, cohort effects, and family effects.

To assess the significance of relatives, we conducted a type III analysis of variance (ANOVA) with the package *car* (Fox & Weisberg, 2019) and extracted pairwise differences in estimated marginal means with the package *emmeans* (Lenth, 2019). We only extracted pairwise differences within focal’s sex and parental SES group: for example, compared having older brothers alive with having no brothers in landless men. For the binomial part of the outcome, the pairwise differences are odds ratios (OR). For the continuous part, the pairwise differences correspond to the estimated difference in age at first marriage in years. All *P*-values were controlled for the Benjamini–Hochberg false discovery rate (FDR) to decrease type I error (Benjamini & Hochberg, 1995).

Plots were built with the package *ggplot2* (Wickham, 2016). In data handling, we utilized the packages *dplyr* (Wickham et al., 2019), *forcats* (Wickham, 2023), and *purrr* (Wickham & Henry, 2023).

#### Number of relatives and children

To simultaneously but separately model lifetime childlessness and the lifetime number of children, we utilized mixed-effects hurdle models to examine the association of relatives with children using the package *glmmTMB* (Brooks et al., 2017; McGillycuddy et al., 2025). We used the number of children (0–20) as a discrete numerical outcome, with remaining childless as the binomial component in the hurdle model. Before fitting models, we ensured adequate sample sizes in both childless and not childless groups across all levels of categorical variables by cross-tabulating.

Similar to the previous models, we first fit full models separately for paternal relatives, maternal relatives, and siblings (also including siblings’ children). These full models included all relatives of the lineage and focal’s sex, parental SES, and birth region as fixed covariates and mother’s identity, birth cohort, and categorical age at first marriage as random effects. We examined model fit visually using the package *DHARMa* (Hartwig, 2022) and assessed multicollinearity with the VIF using the package *car* (Fox & Weisberg, 2019). The models reached good model fit with the zero-truncated Poisson distribution.

As with the marriage models, we performed model selection using the package *MuMIn* (Bartoń, 2025). This model selection was performed separately for the binomial outcome (childless/had children) and the positive discrete outcome (number of children in those who reproduced) due to computational restrictions. Covariates were required to be in all models. We selected the models with the lowest AICc value and fit a hurdle model including all relative types retained in the best-fitting binomial and discrete numerical models. To compare the probability to remain childless and the number of children within each sex and SES class, we also fit three-way interactions between focal’s sex, parental SES, and relatives. The final models included relatives that were selected in model selection (paternal and sibling types, and maternal and sibling types separately) and their three-way interaction with focal’s sex and parental SES, as well as region as a fixed covariate and mother’s identity, birth cohort, and categorical age at first marriage as random effects.

We conducted a type III ANOVA with the package *car* (Fox & Weisberg, 2019) and extracted pairwise differences in estimated marginal means with the package *emmeans* (Lenth, 2019). Similar to the marriage analysis, we only extracted pairwise differences within focal’s sex and parental SES group. For the binomial part of the outcome, the pairwise differences are odds ratios (OR). For the positive discrete part, we transformed ratios of geometric means to percentage differences (Lenth, n.d.), which correspond to the estimated percentage difference in the number of children. All *P*-values were controlled for the Benjamini–Hochberg FDR to decrease type I error (Benjamini & Hochberg, 1995), and plots were built with the package *ggplot2* (Wickham, 2016).

## 3. Results

### 3.1. Number of relatives and marriage

**Figure 1.**
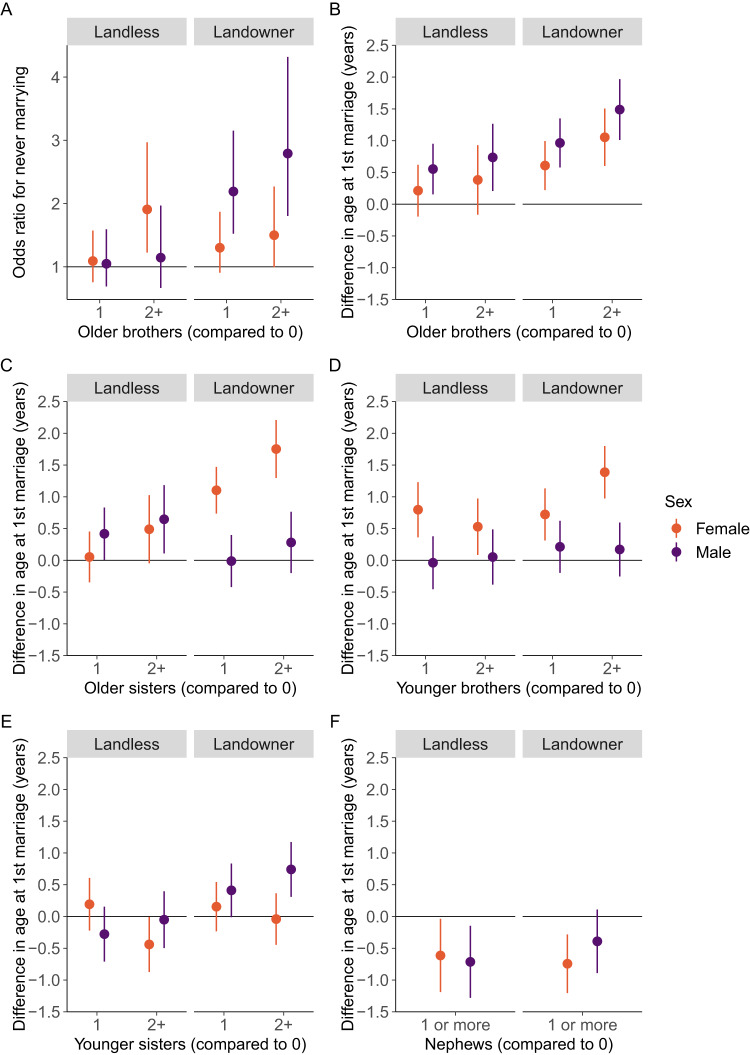
The association of (A) older brothers with never marrying and (B) older brothers, (C) older sisters, (D) younger brothers, (E) younger sisters, and (F) nephews with age at first marriage. Panel (A) represents the odds ratio of never marrying in focals that had one or two or more older brothers alive compared to having none. Other panels represent the difference in age at first marriage in years compared to having no such relative type alive. Points correspond to pairwise comparisons of estimated marginal means between a specific number of a relative type compared to having no such relative alive. Orange points are female focals, and violet points are male focals. Error bars are 95% confidence intervals. Figure 1 long description.

From all relative types, older brothers, older sisters, younger brothers, younger sisters, and nephews were included in the final hurdle model of age at first marriage, including a binomial response for never marrying. After controlling for the FDR, the only relatives whose number at age 15 was associated with the probability of remaining unmarried were older brothers and older sisters. In men from landowning families, having older brothers alive at age 15 was associated with a higher probability to never marry (Figure 1A): the odds of never marrying were 2.2 with one older brother and 2.8 with two or more older brothers compared to having no older brothers. Similarly, in landless women, having two or more older brothers was associated with a higher probability to never marry (OR = 1.9; Figure 1A). Older brothers were not associated with the probability to never marry in landless men or in landowning women. Having one, but not multiple, older sister was associated with a higher probability to never marry in women from landowning families (OR = 1.6).

Age at first marriage was related to having older siblings. Having older brothers alive at age 15 was associated with a higher age at first marriage in men from both landless and landowning families and in women from landowning families (Figure 1B). Landless men with two or more older brothers married, on average, 0.73 years later, landowning men 1.49 years later and landowning women 1.05 years later than those who had no older brothers. Having one older brother compared to having none was also associated with a higher age at first marriage, although the pairwise differences were smaller (Figure 1B). Older brothers were not associated with age at first marriage in women from landless families. After correcting for the FDR, older sisters were only associated with age at first marriage in women from landowning families: having one older sister was associated with 1.10 years higher and having two or more with 1.75 years higher age at first marriage compared to having none (Figure 1C). Older sisters were not associated with age at first marriage in men and women from landless families or in men from landowning families.

Younger siblings were also associated with age at first marriage. In women from landowning families, having one younger brother alive at age 15 was associated with 0.72 years higher age at first marriage compared to having no younger brothers (Figure 1D). Similarly, having two or more younger brothers was associated with 1.39 years higher age at first marriage. In women from landless families, having one, but not multiple, younger brother was associated with 0.80 years higher age at first marriage (Figure 1D). Younger brothers were not associated with age at first marriage in men. Younger sisters were only associated with age at first marriage in men from landowning families: having multiple younger sisters alive at age 15 was associated with 0.74 years higher age at first marriage (Figure 1E). Younger sisters were not associated with age at first marriage in men from landless families or in women.

Nephews were associated with age at first marriage in women from landowning families: those who had at least one nephew alive at age 15 had 0.74 years lower age at first marriage compared to those who had none (Figure 1F). After correcting for the FDR, nephews were not associated with age at first marriage in women from landless families or in men.

ANOVA results are presented in Supplementary Table S4, and all estimates, their confidence limits, and *P*-values in Supplementary Table S5.

### 3.2. Number of relatives and children

**Figure 2.**
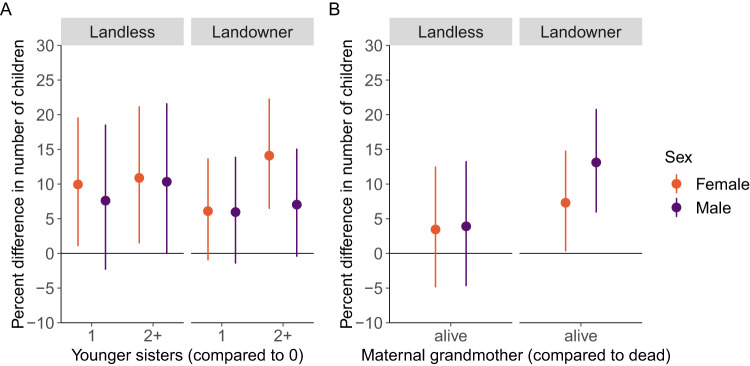
The association of (A) younger sisters and (B) maternal grandmother with the lifetime number of children. The panels represent percent differences in the estimates of the mean lifetime number of children. Points correspond to pairwise comparisons of estimated marginal means between a specific number of relative compared to having no such relative alive. Orange points are female focals, and violet points are male focals. Error bars are 95% confidence intervals. Figure 2 long description.

The final hurdle model of the association of paternal kin with the lifetime number of children and lifetime childlessness included paternal uncles and cousins, younger brothers, and younger sisters. After correcting for the FDR, none of these kin types were associated with lifetime childlessness. Furthermore, after correcting for the FDR, most of these kin types were not associated with the lifetime number of children in those who reproduced at least once. However, younger sisters were associated with the number of children in women from landowning families: having two or more younger sisters alive at age 15 was associated with a 14.1% higher lifetime number of children compared to those having no younger sisters (Figure 2A).

The final model for maternal kin included the maternal grandmother and cousins, younger brothers, younger sisters, and nieces. After correcting for the FDR, none of these kin types were associated with lifetime childlessness. Furthermore, after correcting for the FDR, most of these kin types were not associated with the lifetime number of children in those who reproduced at least once. However, the maternal grandmother was associated with the number of children in men from landowning families: having a maternal grandmother alive at age 15 was associated with a 13.1% higher lifetime number of children in these focals (Figure 2B).

ANOVA can be found in Supplementary Table S7, and all estimates, their confidence limits, and *P*-values can be found in Supplementary Table S8.

## 4. Discussion

In this study, we discovered that kin were associated with marriage and the lifetime number of children, but these associations were highly varied between men and women and between focals from different socioeconomic backgrounds. In summary, having many older or younger siblings alive at age 15 was negatively associated with age at first marriage, whereas younger sisters and the maternal grandmother were positively associated with fertility within marriage. However, these associations were mostly seen for focals from landowning families, with only brothers showing weak associations with age at first marriage also among landless. In contrast, sisters were associated with a higher age at first marriage only in focals from landowning families. Therefore, on average, those without siblings had better chances of marrying during their fertile years. However, these associations also differed between men and women: while having older brothers alive at age 15 was associated with a higher age at first marriage in both sexes, having older sisters and younger brothers was only associated with age at first marriage in women, whereas having younger sisters was associated with age at first marriage in men. In contrast, for women from landowning families, having many younger sisters was associated with higher fertility in marriage, whereas having the maternal grandmother was associated with a higher lifetime number of children in men from landowning families. Although causal conclusions cannot be drawn, these results suggest stronger sibling competition for spouses or resources required for marriage among wealthy focals. Having younger sisters was associated with a higher lifetime number of children, through younger sisters possibly acting as helpers.

We discovered that older siblings were associated with marriage, but not with having children. Having older brothers alive at age 15 was associated with a higher probability to remain unmarried for women from landless families and men from landowning families. Furthermore, having older brothers was associated with a higher age at first marriage for both men and women from landowning families and for men, but not women, from landless families. The association was strongest for men from landowning families. Having older sisters alive at age 15 was associated with a higher probability to remain unmarried and a higher age at first marriage in women from landowning families. Previous studies of historical societies and modern rural patrilineal communities have also discovered that older siblings were associated with a lower likelihood of marriage (Borgerhoff Mulder, 1998; Dribe & Lundh, 2014; Gibson & Gurmu, 2011; Low, 1991; Pettay et al., 2020). However, unlike in some previous studies (Borgerhoff Mulder, 1998; Gibson & Gurmu, 2011; Mace, 1996; Nitsch et al., 2013), older siblings were not associated with the lifetime number of children. It is, however, possible that siblings in later life could have an influence on reproductive success, as we only considered their number when the focal was 15 years of age, providing information on their early life social environment. Sex differences in associations of older siblings with marriage are well explained by same-sex competition between siblings (Borgerhoff Mulder, 1998). Male siblings likely competed for inheritance and resources required to marry. Having many female siblings, on the other hand, may have reduced marriage opportunities and the ability to attract mates (Tymicki, 2004), as communities were small and migration distances were commonly very short (Kauppi et al., 2026; Nitsch et al., 2016). Therefore, it was common to find a spouse within one’s own birth parish. However, with an increasing number of older sisters, it is also possible that focals would receive a smaller dowry (Low, 1991; Mace, 1996), possibly explaining these results as well.

These results on older siblings relating to differences between focals from landless and landowning families may be explained by the inheritance patterns of historical Finland. In landowning families, the oldest son generally received the farm as inheritance and was expected to marry first, whereas younger siblings received, for example, cattle and bedding, and could marry only after the oldest son had married (Moring, 1998). With an increasing number of children, the inheritance divided among siblings decreased accordingly, as most landowners were not particularly wealthy. In contrast, landless families had little to give to their children, and when reaching adulthood, these children had to move out and find work to earn a living (Moring, 1998, 2003), and therefore they may have been less affected by sibling competition (Bengtsson et al., 2014). Previous studies have also discovered that in societies in which resources are transferred intergenerationally, siblings often compete for these resources and marriage opportunities (Gibson & Gurmu, 2011; Mace, 1996). This difference in resource acquisition (inherited compared to gained through working) between the two classes may explain why sibling competition seems to be stronger for the landowning. Previous studies have also discovered that in historical and traditional societies, siblings reduce the likelihood of marrying more in high than in low SES groups (Bengtsson et al., 2014; Gibson & Gurmu, 2011; Pettay et al., 2020). The church and society generally required a man to have a profession or land before marriage, but this requirement was overlooked if the woman was already expecting (Moring, 2003), which was more common for landless than for landowner couples (Moring, 1999). Therefore, it is likely that marriage was not similarly constrained by competition with close family members among landless.

We discovered that having younger siblings alive at age 15 was associated with age at first marriage and with the lifetime number of children, but these associations varied with SES and focal’s sex. Having younger brothers was associated with a higher age at first marriage in women, but more so in women from landowning families. Female focals may postpone their own reproduction to help raise their younger siblings (Emlen, 1995; Low, 1991). Interestingly, having many younger sisters at age 15 was associated with a higher age at first marriage in men, but not in women, from landowning families. However, this association was very small. In landowning women, however, having many younger sisters alive at age 15 was associated with a higher lifetime number of children. One possible reason for this association is that, in this population with natural fertility, younger sisters could act as helpers-in-the-nest, potentially reducing the workload of the parents and thereby increasing fecundity (Sear et al., 2003; Tymicki, 2004). However, it is possible that these results relate to the heritability of fecundity or the healthy family effect (Schaffnit & Sear, 2014). Interestingly, we also discovered that having nephews alive at the focal’s age of 15 was associated with a lower age at first marriage in women from landowning families. Women whose siblings already have children may experience greater social pressure to enter into marriage and reproduce.

Finally, we discovered that having the maternal grandmother alive at age 15 was associated with a higher lifetime number of children in men from landowning families. This result could be an artefact of the healthy family effect (Schaffnit & Sear, 2014), even though we controlled for family effects by including mother’s identity as a random effect. Unlike what we expected, parents were not associated with marriage or the number of children. Parents and their children face reproductive conflict during the years when both are fertile. In patrilocal societies, fathers usually dominate over their sons (Ji et al., 2014), whereas the death of the father can provide landowning people with more opportunities, as the farm and resources were also inherited (Moring, 1998, 1999). It is possible that in our study system, the father’s presence does not constrain marriage opportunities, or that our analytical approach did not detect this possible link. Interestingly, we did not find any associations between fertility outcomes and paternal relatives. It is possible that our relatively limited sample size did not allow us to detect these associations. Before correcting for the FDR, paternal cousins showed a small association with lifetime childlessness and paternal uncles with age at first marriage. However, these weak associations became non-significant after the correction. However, it is also possible that during our long study period, the social environment, such as the strength of patrilocality, changed, or that the environment differs between the diverse regions of Finland, limiting the detection of significant associations. In historical Finland, both paternal and maternal kin tended to live nearby, as dispersal distances were short (Kauppi et al., 2026). Future studies should examine the association of paternal kin with fertility outcomes in more detail, with a focus also on the distance to kin, not only their presence.

After correcting for the FDR, we did not find any significant associations of relatives with lifetime childlessness. In our previous study, childlessness was highly linked to age at first marriage (Salonen et al., 2024), and it is possible that the variation remaining after controlling for age at first marriage was not large enough to detect kin effects. Furthermore, the associations of kin with the lifetime number of children were much more modest than the associations of kin with age at first marriage. It is possible that having kin, for example, siblings alive at the time of reproduction, would be more important than the early social environment. However, it is also possible that the harmful effects of siblings on fertility in previous studies may be moderated by a later age at first marriage.

Previous studies have not examined the associations between extended family members and fertility, but some studies have discovered that aunts, uncles, and cousins can influence child survival (Borgerhoff Mulder, 2007; Hadley, 2004; Lahdenperä et al., 2025; Nitsch et al., 2014). However, in our sample of historical Finns, these extended family members were not associated with fertility outcomes.

Based on previous research, we formulated several hypotheses and found support for some of them. Firstly, we hypothesized that having close relatives alive at age 15 would be more strongly associated with fertility outcomes than having more distantly related relatives alive. This was indeed the case, with only the maternal grandmother and nephews being significantly associated with our fertility outcomes, whereas many sibling types were associated with these outcomes. We formulated two alternative hypotheses, predicting that the association of close relatives with fertility outcomes could be either more positive (first alternative hypothesis) or more negative (second alternative hypothesis) than the association of extended family members. The former hypothesis is based on the kin selection theory: close relatives provide more help, as they can gain more inclusive fitness from helping (Hamilton, 1964). Contrasting this theory and supporting our second alternative hypothesis, our results may indicate competition for inheritance, potential spouses, or other resources between siblings. Secondly, we also hypothesized that vertical relatives (parents and grandparents) would be more positively associated with fertility outcomes than horizontal relatives (siblings and cousins). This hypothesis was only partially supported. While we found that having the maternal grandmother alive at age 15 was positively associated with the lifetime number of children, other vertical relatives did not relate to our fertility outcomes. Thirdly, we hypothesized that maternal relatives would be more positively associated with fertility outcomes than paternal relatives. Indeed, the maternal grandmother was the only extended family member (apart from nephews) associated with fertility, while, after correcting for the FDR, paternal family members did not associate with the outcomes. Fourthly, we hypothesized that kin would be more strongly associated with fertility outcomes in focals from landowning families than in focals from landless families. We indeed discovered that while siblings had few associations with fertility outcomes in landless focals, their associations were much stronger in focals from landowning families. We formulated two alternative hypotheses, predicting two alternative scenarios. Our first alternative hypothesis, supported by theories predicting greater cooperation under resource abundance (Boyd, 1982; Frank, 1998) and some empirical evidence (human population: Schaffnit & Sear, 2014), predicted that kin would be more positively associated with fertility among the wealthy than among the poor. Our second alternative hypothesis suggested that the association of kin with fertility outcomes would be more negative among the wealthy than among the poor, as the wealthy can compete for intergenerationally transmitted resources (Gibson & Gurmu, 2011; Mace, 1996; Moring, 1998). We found support for this second alternative. Therefore, in this sample of historical Finns, a high level of resources may intensify competition between relatives. Fifthly, we hypothesized that kin may have contrasting associations with the different fertility outcomes. Indeed, we discovered that kin were mostly associated with either marriage or the number of children, not both, and that while younger sisters were associated with a higher age at first marriage, they were also associated with a higher lifetime number of children. These associations between kin and fertility outcomes likely differ between populations and societies, as, for example, subsistence strategy, marriage systems, inheritance patterns, and residence patterns likely influence kin effects (Sear & Mace, 2008).

This study has limitations. We did not consider where the kin resided, only whether they were alive, independent of their proximity. The proximity of kin and the actual childcare provided may be important factors for fertility (Hacker et al., 2021; Rutigliano, 2023; Snopkowski & Sear, 2016; Willführ et al., 2022). However, the traditional Finnish society was patrilocal, meaning that paternal kin usually lived nearby (Moring, 1999), and in historical societies, migration was mostly over short distances (Kauppi et al., 2026; Pooley & Turnbull, 1998). Therefore, it is likely that kin, especially paternal kin, lived close to the focals. Furthermore, we studied the lifetime number of children instead of age-specific fertility in a sample of individuals who needed to survive until relatively old age. However, the lifetime number of children itself is interesting, as it highlights a large investment in reproduction and is more related to population-level patterns of fertility. Finally, reproductive success and interbirth interval are moderately heritable (Pettay et al., 2008, 2005), and family size can be transmitted over generations even to collateral kin (Kolk, 2014), and therefore, a positive association of kin alive with the lifetime number of children can be influenced by this transmission. Our study also has many strengths. Firstly, the long time period, along with the meticulously collected data, allows for a reliable study of marriage and fertility. Secondly, our dataset consists of family lineages, and this allowed us, for the first time, to assess the link between aunts, uncles, and cousins and fertility, and revealed that even some more distant kin can influence fertility. Thirdly, the possibility of assessing the level of available resources based on the occupation of the focal individual’s parents provides a relatively clear view of the SES in historical Finland.

## 5. Conclusions

The aim of the present study was to examine the association of close and more distant kin with fertility outcomes in the historical agrarian Finland. We studied how having kin alive at the age of 15 years was associated with the probability to be married, the age at first marriage, the probability to remain childless, and the lifetime number of children in those who reproduced. Among the landless, kin had few associations with marriage outcomes. However, older brothers in men and younger brothers in women were associated with a higher age at first marriage. For the landowning, kin were associated with marriage and the lifetime number of children differently: older siblings were associated with a lower probability to marry and a higher age at first marriage, while maternal grandmother for men and younger sisters for women were linked to a higher number of children. In conclusion, sibling competition for, for example, inheritance may be important for fertility, while extended family members alive at the age of 15 years play only a small role. These findings suggest that the level of available resources and the focal’s sex can influence kin–fertility associations, and that these associations are not necessarily uniform across different fertility outcomes. This study contributes to our understanding of kin influences on fertility and adds to a body of research indicating that children of landowning families, who receive inheritance from their parents, face stronger sibling competition than landless. Broadly, understanding the fertility effects of relatives, and how these effects are modulated by resources and sex, can provide insights into the evolution of human sociality, cooperative breeding, and reproductive strategies.

## Supporting information

10.1017/ehs.2026.10060.sm001 Salonen et al. supplementary material Salonen et al. supplementary material

## Supplementary material

The supplementary material for this article can be found at https://doi.org/10.1017/ehs.2026.10060.

## Research transparency and reproducibility interest

The data and code used are available as Supplementary Material.

## Figure 1 Long description

The image A showing a point and error bar plot split into Landless and Landowner. The vertical axis label is Odds ratio for never marrying, with tick labels 1, 2, 3, 4. The horizontal axis label is Older brothers (compared to 0), with category labels 1 and 2 plus in each section. A horizontal reference line is drawn at 1. A legend in the figure labels Sex with Female and Male and each category shows one Female point and one Male point with vertical error bars. Landless, category 1: Female near 1.0; Male near 1.0. Landless, category 2 plus: Female near 2.0; Male near 1.1. Landowner, category 1: Female near 1.3; Male near 2.2. Landowner, category 2 plus: Female near 1.5; Male near 2.8. Pattern described by the plotted positions: in Landowner, Male points are higher than Female at both 1 and 2 plus; in Landless, the Female point at 2 plus is higher than the Male point. The image B showing a point and error bar plot split into Landless and Landowner. The vertical axis label is Difference in age at 1st marriage (years), with tick labels negative 1.5, negative 1.0, negative 0.5, 0.0, 0.5, 1.0, 1.5, 2.0, 2.5. The horizontal axis label is Older brothers (compared to 0), with category labels 1 and 2 plus in each section. A horizontal reference line is drawn at 0.0. Each category shows one Female point and one Male point with vertical error bars. Landless, category 1: Female near 0.2; Male near 0.6. Landless, category 2 plus: Female near 0.4; Male near 0.8. Landowner, category 1: Female near 0.6; Male near 1.0. Landowner, category 2 plus: Female near 1.1; Male near 1.5. Pattern described by the plotted positions: values are above 0.0 in all shown categories; Male points are higher than Female points in both Landless and Landowner; 2 plus is higher than 1 within each land status for both Female and Male. The image C showing a point and error bar plot split into Landless and Landowner. The vertical axis label is Difference in age at 1st marriage (years), with tick labels negative 1.5, negative 1.0, negative 0.5, 0.0, 0.5, 1.0, 1.5, 2.0, 2.5. The horizontal axis label is Older sisters (compared to 0), with category labels 1 and 2 plus in each section. A horizontal reference line is drawn at 0.0. Each category shows one Female point and one Male point with vertical error bars. Landless, category 1: Female near 0.1; Male near 0.4. Landless, category 2 plus: Female near 0.5; Male near 0.7. Landowner, category 1: Female near 1.1; Male near 0.0. Landowner, category 2 plus: Female near 1.8; Male near 0.3. Pattern described by the plotted positions: Female points are higher than Male points in Landowner at both 1 and 2 plus; in Landless, Male points are higher than Female points at both 1 and 2 plus; Female values increase from 1 to 2 plus in both Landless and Landowner. The image D showing a point and error bar plot split into Landless and Landowner. The vertical axis label is Difference in age at 1st marriage (years), with tick labels negative 1.5, negative 1.0, negative 0.5, 0.0, 0.5, 1.0, 1.5, 2.0, 2.5. The horizontal axis label is Younger brothers (compared to 0), with category labels 1 and 2 plus in each section. A horizontal reference line is drawn at 0.0. Each category shows one Female point and one Male point with vertical error bars. Landless, category 1: Female near 0.8; Male near 0.0. Landless, category 2 plus: Female near 0.6; Male near 0.1. Landowner, category 1: Female near 0.7; Male near 0.2. Landowner, category 2 plus: Female near 1.4; Male near 0.2. Pattern described by the plotted positions: Female points are above Male points in all shown categories; Female values are above 0.0 in all shown categories; Male values are near 0.0 to 0.2; Landowner Female at 2 plus is higher than Landowner Female at 1. The image E showing a point and error bar plot split into Landless and Landowner. The vertical axis label is Difference in age at 1st marriage (years), with tick labels negative 1.5, negative 1.0, negative 0.5, 0.0, 0.5, 1.0, 1.5, 2.0, 2.5. The horizontal axis label is Younger sisters (compared to 0), with category labels 1 and 2 plus in each section. A horizontal reference line is drawn at 0.0. Each category shows one Female point and one Male point with vertical error bars. Landless, category 1: Female near 0.2; Male near negative 0.3. Landless, category 2 plus: Female near negative 0.5; Male near 0.0. Landowner, category 1: Female near 0.2; Male near 0.4. Landowner, category 2 plus: Female near 0.0; Male near 0.8. Pattern described by the plotted positions: Landless Female changes from above 0.0 at 1 to below 0.0 at 2 plus; Landowner Male increases from about 0.4 at 1 to about 0.8 at 2 plus. The image F showing a point and error bar plot split into Landless and Landowner. The vertical axis label is Difference in age at 1st marriage (years), with tick labels negative 1.5, negative 1.0, negative 0.5, 0.0, 0.5, 1.0, 1.5, 2.0, 2.5. The horizontal axis label is Nephews (compared to 0), with category label 1 or more in each section. A horizontal reference line is drawn at 0.0. Each category shows one Female point and one Male point with vertical error bars. Landless, 1 or more: Female near negative 0.6; Male near negative 0.7. Landowner, 1 or more: Female near negative 0.8; Male near negative 0.4. Pattern described by the plotted positions: all shown values are below 0.0; in Landowner, Male is higher than Female; in Landless, Female and Male are close together. Across images A to F, the same stratification is used: each plot is split into Landless and Landowner and each category includes one Female point and one Male point with error bars. Image A uses Odds ratio for never marrying on the vertical axis with a reference line at 1, while images B to F use Difference in age at 1st marriage (years) on the vertical axis with a reference line at 0.0.

Navigate back to Figure 1.

## Figure 2 Long description

The image A showing two dot and error bar plots labeled Landless and Landowner. Landless plot. Horizontal axis label: Younger sisters (compared to 0). Categories: 1, 2 plus. Vertical axis label: Percent difference in number of children. Range: negative 10 to 30. Legend label: Sex. Series labels: Female, Male. Female coordinate pairs: (1, 10), (2 plus, 11). Male coordinate pairs: (1, 7), (2 plus, 10). Landowner plot. Horizontal axis label: Younger sisters (compared to 0). Categories: 1, 2 plus. Vertical axis label: Percent difference in number of children. Range: negative 10 to 30. Female coordinate pairs: (1, 6), (2 plus, 14). Male coordinate pairs: (1, 6), (2 plus, 7). The image B showing two dot and error bar plots labeled Landless and Landowner. Landless plot. Horizontal axis label: Maternal grandmother (compared to dead). Category: alive. Vertical axis label: Percent difference in number of children. Range: negative 10 to 30. Legend label: Sex. Series labels: Female, Male. Female coordinate pairs: (alive, 3). Male coordinate pairs: (alive, 4). Landowner plot. Horizontal axis label: Maternal grandmother (compared to dead). Category: alive. Vertical axis label: Percent difference in number of children. Range: negative 10 to 30. Female coordinate pairs: (alive, 7). Male coordinate pairs: (alive, 13).

Navigate back to Figure 2.
