# Supplementary material for: Resources modify the associations between kin and fertility in historical Finland
Source: Evol Hum Sci. 2026 Jul 3;8:e30. doi: 10.1017/ehs.2026.10060 (PMC13435151; doi:10.1017/ehs.2026.10060)
Supplement: Supplementary file 1 [file S2513843X26100607sup001.zip › r-script.docx]

R script

## **Packages**

```
# for data editing etc.
library(dplyr)
library(tidyr)

# for glmm
library(glmmTMB)

# for model fit
library(DHARMa)
library(performance)

# for anova
library(car)

# for pairwise comparisons
library(emmeans)

# for plotting
library(ggplot2)

# for combining plots into panels
library(patchwork)
```

## **Data**

- ID: the unique id of the focal individual
- sex: focal’s sex: 1 = male, 2 = female
- mumid: the unique id of the focal’s mother
- cohort: 10-year birth cohort (factor)
- SES.class: focal’s parents’ combined social class. “landowner” = wealthy (owns their land or otherwise wealthy), “landless” = average or poor, with no land or education
- four.region: the three-level region where focal was born in
- evermarried: whether focal was married. 1 = yes, 0 = no
- mar1age: age at first marriage
- mar1age.zi: age at first marriage in years including 0s for those who did not marry
- allkidsknown: whether the focal survived until 45 with their full life history known, 1 = yes, 0 = no
- kids: number of children
- father, mother, father.father, father.mother, mother.father, mother.mother: whether this kin was alive at focal’s age 15, 1 = yes, 0 = no
- older.sisters to nephews: categorized number of these kin alive at focal’s age 15

```
# read in data
child.data.final <- read.table(file="data-for-publication.csv")

# categorize numeric that are actually factors
child.data.final <- child.data.final %>%
  mutate_if(is.character,as.factor) %>%
  mutate(ID = as.factor(ID),
         mumid = as.factor(mumid),
         four.region = as.factor(four.region),
         father = as.factor(father),
         mother = as.factor(mother),
         father.father = as.factor(father.father),
         father.mother = as.factor(father.mother),
         mother.father = as.factor(mother.father),
         mother.mother = as.factor(mother.mother),
         sex = as.factor(sex))
```

## **Marriage**

### **Crosstabulation**

```
# crosstabulation of marriage probability and relatives separately for landless and landowner families

child.data.landless <- child.data.final %>%
  filter(SES.class == "landless")

table(child.data.landless$mother,child.data.landless$evermarried)
table(child.data.landless$father,child.data.landless$evermarried)
table(child.data.landless$father.mother,child.data.landless$evermarried)
table(child.data.landless$father.father,child.data.landless$evermarried)
table(child.data.landless$mother.mother,child.data.landless$evermarried)
table(child.data.landless$mother.father,child.data.landless$evermarried)

table(child.data.landless$older.brothers,child.data.landless$evermarried)
table(child.data.landless$older.sisters,child.data.landless$evermarried)
table(child.data.landless$younger.brothers,child.data.landless$evermarried)
table(child.data.landless$younger.sisters,child.data.landless$evermarried)

table(child.data.landless$father.sister,child.data.landless$evermarried)
table(child.data.landless$father.brother,child.data.landless$evermarried)
table(child.data.landless$mother.sister,child.data.landless$evermarried)
table(child.data.landless$mother.brother,child.data.landless$evermarried)

table(child.data.landless$pat.cousins,child.data.landless$evermarried)
table(child.data.landless$mat.cousins,child.data.landless$evermarried)

table(child.data.landless$nieces,child.data.landless$evermarried)
table(child.data.landless$nephews,child.data.landless$evermarried)


# landowner families
child.data.landed <- child.data.final %>%
  filter(SES.class == "landowner")

table(child.data.landed$mother,child.data.landed$evermarried)
table(child.data.landed$father,child.data.landed$evermarried)
table(child.data.landed$father.mother,child.data.landed$evermarried)
table(child.data.landed$father.father,child.data.landed$evermarried)
table(child.data.landed$mother.mother,child.data.landed$evermarried)
table(child.data.landed$mother.father,child.data.landed$evermarried)

table(child.data.landed$older.brothers,child.data.landed$evermarried)
table(child.data.landed$older.sisters,child.data.landed$evermarried)
table(child.data.landed$younger.brothers,child.data.landed$evermarried)
table(child.data.landed$younger.sisters,child.data.landed$evermarried)

table(child.data.landed$father.sister,child.data.landed$evermarried)
table(child.data.landed$father.brother,child.data.landed$evermarried)
table(child.data.landed$mother.sister,child.data.landed$evermarried)
table(child.data.landed$mother.brother,child.data.landed$evermarried)

table(child.data.landed$pat.cousins,child.data.landed$evermarried)
table(child.data.landed$mat.cousins,child.data.landed$evermarried)

table(child.data.landed$nieces,child.data.landed$evermarried)
table(child.data.landed$nephews,child.data.landed$evermarried)

#sample size enough for both
```

### **Full model, paternal**

first create data with no missing information except in mar1age

```
marriage.data.paternal <- child.data.final %>%
  dplyr::select(ID, evermarried, mar1age, mar1age.zi, sex, cohort, four.region,
                SES.class,mumid, father, father.father, father.mother,
                father.brother, father.sister, pat.cousins) %>%
  drop_na(!mar1age) %>%
  droplevels()
```

full model with region, sex and SES.class as fixed covariates paternal relatives also mumid and cohort as random variables

```
full.paternal <- glmmTMB(mar1age.zi ~ four.region + sex + SES.class +
                     father + father.father + father.mother +
                       father.brother + father.sister + pat.cousins +
                       (1|mumid) + (1|cohort),
                     ziformula = ~.,
                     dispformula = ~ 1,
                   data = marriage.data.paternal,
                   family= gaussian(link="identity"))
```

model fit

```
sim.paternal <- simulateResiduals(fittedModel = full.paternal, plot = T)
# some  slight trend in the residual plot
# some underdispersion
testDispersion(sim.paternal)

# categorical variables
# all groups seem to have equal variance
testCategorical(sim.paternal, catPred = marriage.data.paternal$sex)
testCategorical(sim.paternal, catPred = marriage.data.paternal$four.region)
testCategorical(sim.paternal, catPred = marriage.data.paternal$SES.class)
testCategorical(sim.paternal, catPred = marriage.data.paternal$father)
testCategorical(sim.paternal, catPred = marriage.data.paternal$father.father)
testCategorical(sim.paternal, catPred = marriage.data.paternal$father.mother)
testCategorical(sim.paternal, catPred = marriage.data.paternal$father.brother)
testCategorical(sim.paternal, catPred = marriage.data.paternal$father.sister)
testCategorical(sim.paternal, catPred = marriage.data.paternal$pat.cousins)

# recalculate residuals by grouping by mumid
# this could show over or underdispersion
sim.paternal.2 <- recalculateResiduals(sim.paternal,
                                       group=marriage.data.paternal$mumid)
plot(sim.paternal.2)
# some underdispersion
# underdispersion is a smaller problem than overdispersion
testDispersion(sim.paternal.2)

# compare to model with Gamma distribution
full.paternal.gamma <- glmmTMB(mar1age.zi ~ four.region + sex + SES.class +
                           father + father.father + father.mother +
                           father.brother + father.sister + pat.cousins +
                             (1|mumid) + (1|cohort),
                         ziformula = ~.,
                         data = marriage.data.paternal,
                         control = glmmTMBControl(optCtrl = list(iter.max=5000, eval.max=1000)),
                         family= ziGamma(link="log"))

sim.paternal.gamma <- simulateResiduals(fittedModel = full.paternal.gamma,
                                        plot = T)
testDispersion(sim.paternal.gamma)
sim.paternal.gamma.2 <- recalculateResiduals(sim.paternal.gamma, group=marriage.data.paternal$mumid)
testDispersion(sim.paternal.gamma.2)
# underdispersion is not any better

# compare to model with lognormal distribution
full.paternal.log <- glmmTMB(mar1age.zi ~ four.region + sex + SES.class +
                                 father + father.father + father.mother +
                                 father.brother + father.sister + pat.cousins +
                               (1|mumid) + (1|cohort),
                               ziformula = ~.,
                               data = marriage.data.paternal,
                               control = glmmTMBControl(optCtrl =
                                                          list(iter.max=5000,
                                                               eval.max=1000)),
                               family= lognormal(link="log"))

sim.paternal.log <- simulateResiduals(fittedModel = full.paternal.log,
                                      plot = T)
# some underdispersion
testDispersion(sim.paternal.log)
sim.paternal.log.2 <- recalculateResiduals(sim.paternal.log, group=marriage.data.paternal$mumid)
testDispersion(sim.paternal.log.2)
# doesn't fix underdispersion
# otherwise models look good, so let’s stick to gaussian
```

multicollinearity

check_collinearity(full.paternal, component = c('all'))

### **Model selection, paternal**

select best-fitting model for paternal kin region, sex and SES class will be kept in all models paternal kin allowed to vary

because of computational limitations, binomial and continuous part of the outcome run separately

```
# binomial part
full.paternal.bin <- glmmTMB(evermarried ~ four.region + sex + SES.class +
                               father + father.father + father.mother +
                               father.brother + father.sister + pat.cousins +
                               (1|mumid) + (1|cohort),
                             data = marriage.data.paternal,
                             family= binomial(link="logit"))

# option to prevent fitting models to different datasets
options(na.action = "na.fail")

# model selection
select.paternal.bin <- dredge(full.paternal.bin,
                              fixed = c("cond(four.region)", "cond(sex)",
                                        "cond(SES.class)"),
                              trace = 100, rank="AICc")
select.paternal.bin


# continuous part
# have to remove rows with missing mar1age
marriage.data.paternal.2 <- marriage.data.paternal %>%
  filter(!is.na(mar1age))

full.paternal.cont <- glmmTMB(mar1age ~ four.region + sex + SES.class +
                                father + father.father + father.mother +
                                father.brother + father.sister + pat.cousins +
                                (1|mumid) + (1|cohort),
                              data = marriage.data.paternal.2,
                              family= gaussian(link="identity"))

# option to prevent fitting models to different datasets
options(na.action = "na.fail")

# model selection
select.paternal.cont <- dredge(full.paternal.cont, fixed =
                                 c("cond(four.region)", "cond(sex)",
                                   "cond(SES.class)"),
                               trace = 100, rank="AICc")
select.paternal.cont
```

### **Full model, maternal**

first create data with no missing information except in mar1age

```
marriage.data.maternal <- child.data.final %>%
  dplyr::select(ID, evermarried, mar1age, mar1age.zi, sex, cohort, four.region,
                SES.class, mumid,mother, mother.father, mother.mother,
                mother.brother, mother.sister, mat.cousins) %>%
  drop_na(!mar1age) %>%
  droplevels()
```

full model with region, sex and SES.class as fixed covariates maternal relatives also mumid and cohort as random variables

```
full.maternal <- glmmTMB(mar1age.zi ~ four.region + sex + SES.class +
                           mother + mother.father + mother.mother +
                           mother.brother + mother.sister + mat.cousins +
                           (1|mumid) + (1|cohort),
                         ziformula = ~.,
                         dispformula = ~ 1,
                         data = marriage.data.maternal,
                         family= gaussian(link="identity"))
```

model fit

```
sim.maternal <- simulateResiduals(fittedModel = full.maternal, plot = T)
# some  slight trend in the residual plot
# some underdispersion
testDispersion(sim.maternal)

# categorical variables
# all groups seem to have equal variance
testCategorical(sim.maternal, catPred = marriage.data.maternal$sex)
testCategorical(sim.maternal, catPred = marriage.data.maternal$four.region)
testCategorical(sim.maternal, catPred = marriage.data.maternal$SES.class)
testCategorical(sim.maternal, catPred = marriage.data.maternal$mother)
testCategorical(sim.maternal, catPred = marriage.data.maternal$mother.father)
testCategorical(sim.maternal, catPred = marriage.data.maternal$mother.mother)
testCategorical(sim.maternal, catPred = marriage.data.maternal$mother.brother)
testCategorical(sim.maternal, catPred = marriage.data.maternal$mother.sister)
testCategorical(sim.maternal, catPred = marriage.data.maternal$mat.cousins)

# recalculate residuals by grouping by mumid
# this could show over or underdispersion
sim.maternal.2 <- recalculateResiduals(sim.maternal,
                                       group=marriage.data.maternal$mumid)
plot(sim.maternal.2)
# some underdispersion
# underdispersion is a smaller problem than overdispersion
testDispersion(sim.maternal.2)


# compare to model with Gamma distribution
full.maternal.gamma <- glmmTMB(mar1age.zi ~ four.region + sex + SES.class +
                                 mother + mother.father + mother.mother +
                                 mother.brother + mother.sister + mat.cousins +
                                 (1|mumid) + (1|cohort),
                               ziformula = ~.,
                               dispformula = ~ 1,
                               data = marriage.data.maternal,
                               control = glmmTMBControl(optCtrl =
                                                          list(iter.max=5000,
                                                               eval.max=1000)),
                               family= ziGamma(link="log"))

sim.maternal.gamma <- simulateResiduals(fittedModel = full.maternal.gamma,
                                        plot = T)
testDispersion(sim.maternal.gamma)
sim.maternal.gamma.2 <- recalculateResiduals(sim.maternal.gamma, group=marriage.data.maternal$mumid)
testDispersion(sim.maternal.gamma.2)
# very similar underdispersion


# compare to model with lognormal distribution
full.maternal.log <- glmmTMB(mar1age.zi ~ four.region + sex + SES.class +
                               mother + mother.father + mother.mother +
                               mother.brother + mother.sister + mat.cousins +
                               (1|mumid) + (1|cohort),
                             ziformula = ~.,
                             dispformula = ~ 1,
                             data = marriage.data.maternal,
                             control = glmmTMBControl(optCtrl =
                                                        list(iter.max=5000,
                                                             eval.max=1000)),
                             family= lognormal(link="log"))

sim.maternal.log <- simulateResiduals(fittedModel = full.maternal.log, plot = T)
# some underdispersion
testDispersion(sim.maternal.log)
sim.maternal.log.2 <- recalculateResiduals(sim.maternal.log, group=marriage.data.maternal$mumid)
testDispersion(sim.maternal.log.2)
# doesn't improve dispersion
# otherwise models look good, so let's stick to gaussian
```

multicollinearity

check_collinearity(full.maternal, component = c('all'))

### **Model selection, maternal**

select best-fitting model for maternal kin region, sex and SES class will be kept in all models maternal kin allowed to vary

because of computational limitations, binomial and continuous part of the outcome run separately

```
# binomial part
full.maternal.bin <- glmmTMB(evermarried ~ four.region + sex + SES.class +
                               mother + mother.father + mother.mother +
                               mother.brother + mother.sister + mat.cousins +
                               (1|mumid) + (1|cohort),
                             data = marriage.data.maternal,
                             family= binomial(link="logit"))

# option to prevent fitting models to different datasets
options(na.action = "na.fail")

# model selection
select.maternal.bin <- dredge(full.maternal.bin,
                              fixed = c("cond(four.region)", "cond(sex)",
                                        "cond(SES.class)"),
                              trace = 100, rank="AICc")
select.maternal.bin


# continuous part
# have to remove rows with missing mar1age
marriage.data.maternal.2 <- marriage.data.maternal %>%
  filter(!is.na(mar1age))

full.maternal.cont <- glmmTMB(mar1age ~ four.region + sex + SES.class +
                                mother + mother.father + mother.mother +
                                mother.brother + mother.sister + mat.cousins +
                                (1|mumid) + (1|cohort),
                              data = marriage.data.maternal.2,
                              family= gaussian(link="identity"))

# option to prevent fitting models to different datasets
options(na.action = "na.fail")

# model selection
select.maternal.cont <- dredge(full.maternal.cont,
                               fixed = c("cond(four.region)", "cond(sex)",
                                         "cond(SES.class)"),
                               trace = 100, rank="AICc")
select.maternal.cont
```

### **Full model, siblings**

first create data with no missing information except in mar1age

```
marriage.data.sibnib <- child.data.final %>%
  dplyr::select(ID, evermarried, mar1age, mar1age.zi, sex, cohort, four.region,
                SES.class, mumid,older.brothers, older.sisters,
                younger.brothers, younger.sisters, nephews, nieces) %>%
  drop_na(!mar1age) %>%
  droplevels()
```

full model with region, sex and SES.class as fixed covariates siblings & niblings also mumid and cohort as random variables

```
full.sibnib <- glmmTMB(mar1age.zi ~ four.region + sex + SES.class +
                         older.brothers + older.sisters + younger.brothers +
                         younger.sisters + nephews + nieces +
                         (1|mumid) + (1|cohort),
                         ziformula = ~.,
                         dispformula = ~ 1,
                         data = marriage.data.sibnib,
                         family= gaussian(link="identity"))
```

model fit

```
sim.sibnib <- simulateResiduals(fittedModel = full.sibnib, plot = T)
# some underdispersion
testDispersion(sim.sibnib)

# categorical variables
# all groups seem to have equal variance
testCategorical(sim.sibnib, catPred = marriage.data.sibnib$sex)
testCategorical(sim.sibnib, catPred = marriage.data.sibnib$four.region)
testCategorical(sim.sibnib, catPred = marriage.data.sibnib$SES.class)
testCategorical(sim.sibnib, catPred = marriage.data.sibnib$older.brothers)
testCategorical(sim.sibnib, catPred = marriage.data.sibnib$older.sisters)
testCategorical(sim.sibnib, catPred = marriage.data.sibnib$younger.brothers)
testCategorical(sim.sibnib, catPred = marriage.data.sibnib$younger.sisters)
testCategorical(sim.sibnib, catPred = marriage.data.sibnib$nephews)
testCategorical(sim.sibnib, catPred = marriage.data.sibnib$nieces)

# recalculate residuals by grouping by mumid
# this could show over or underdispersion
sim.sibnib.2 <- recalculateResiduals(sim.sibnib,
                                     group=marriage.data.sibnib$mumid)
plot(sim.sibnib.2)
# some underdispersion
# underdispersion is a smaller problem than overdispersion
testDispersion(sim.sibnib.2)


# compare to model with Gamma distribution
full.sibnib.gamma <- glmmTMB(mar1age.zi ~ four.region + sex + SES.class +
                                 older.brothers + older.sisters +
                               younger.brothers + younger.sisters +
                                 nephews + nieces + (1|mumid) + (1|cohort),
                               ziformula = ~.,
                               dispformula = ~ 1,
                               data = marriage.data.sibnib,
                               control = glmmTMBControl(optCtrl =
                                                          list(iter.max=5000,
                                                               eval.max=1000)),
                               family= ziGamma(link="log"))

sim.sibnib.gamma <- simulateResiduals(fittedModel = full.sibnib.gamma, plot = T)
testDispersion(sim.sibnib.gamma)
sim.sibnib.gamma.2 <- recalculateResiduals(sim.sibnib.gamma, group=marriage.data.sibnib$mumid)
testDispersion(sim.sibnib.gamma.2)
# very similar underdispersion


# compare to model with lognormal distribution
full.sibnib.log <- glmmTMB(mar1age.zi ~ four.region + sex + SES.class +
                               older.brothers + older.sisters +
                             younger.brothers + younger.sisters +
                               nephews + nieces + (1|mumid) + (1|cohort),
                             ziformula = ~.,
                             dispformula = ~ 1,
                             data = marriage.data.sibnib,
                             control = glmmTMBControl(optCtrl =
                                                        list(iter.max=5000,
                                                             eval.max=1000)),
                             family= lognormal(link="log"))

sim.sibnib.log <- simulateResiduals(fittedModel = full.sibnib.log, plot = T)
# some underdispersion
testDispersion(sim.sibnib.log)
sim.sibnib.log.2 <- recalculateResiduals(sim.sibnib.log,
                                         group=marriage.data.sibnib$mumid)
testDispersion(sim.sibnib.log.2)
# doesn't improve dispersion
# otherwise models look good, so let's stick to gaussian
```

multicollinearity

check_collinearity(full.sibnib, component = c('all'))

### **Model selection, siblings**

select best-fitting model for siblings & niblings region, sex and SES class will be kept in all models siblings & niblings allowed to vary

because of computational limitations, binomial and continuous part of the outcome run separately

```
# binomial part
full.sibnib.bin <- glmmTMB(evermarried ~ four.region + sex + SES.class +
                             older.brothers + older.sisters +
                             younger.brothers + younger.sisters +
                             nephews + nieces + (1|mumid) + (1|cohort),
                             data = marriage.data.sibnib,
                             family= binomial(link="logit"))

# option to prevent fitting models to different datasets
options(na.action = "na.fail")

# model selection
select.sibnib.bin <- dredge(full.sibnib.bin,
                            fixed = c("cond(four.region)", "cond(sex)",
                                      "cond(SES.class)"),
                              trace = 100, rank="AICc")
select.sibnib.bin


# continuous part
# have to remove rows with missing mar1age

marriage.data.sibnib.2 <- marriage.data.sibnib %>%
  filter(!is.na(mar1age))

full.sibnib.cont <- glmmTMB(mar1age ~ four.region + sex + SES.class +
                              older.brothers + older.sisters +
                              younger.brothers + younger.sisters +
                              nephews + nieces + (1|mumid) + (1|cohort),
                              data = marriage.data.sibnib.2,
                              family= gaussian(link="identity"))

# option to prevent fitting models to different datasets
options(na.action = "na.fail")

# model selection
select.sibnib.cont <- dredge(full.sibnib.cont,
                             fixed = c("cond(four.region)", "cond(sex)",
                                       "cond(SES.class)"),
                               trace = 100, rank="AICc")
select.sibnib.cont
```

### **Model selection, paternal and siblings**

only those relatives who were selected in their respective models paternal: father, father’s sister, paternal cousins sibling: nephews, all sibling types

```
marriage.data.patsib <- child.data.final %>%
  dplyr::select(ID, evermarried, mar1age, mar1age.zi, sex, cohort, four.region,
                SES.class, mumid,father, father.sister, pat.cousins,
                older.brothers, older.sisters, younger.brothers,
                younger.sisters,nephews) %>%
  drop_na(!mar1age) %>%
  droplevels()

# select best-fitting model for paternal kin + siblings
# region, sex and SES class will be kept in all models
# paternal kin + siblings allowed to vary

# because of computational limitations, binomial and continuous part of the outcome run separately

full.patsib.bin <- glmmTMB(evermarried ~ four.region + sex + SES.class +
                             father + father.sister + pat.cousins +
                             older.brothers + older.sisters +
                             younger.brothers + younger.sisters +
                             nephews + (1|mumid) + (1|cohort),
                           data = marriage.data.patsib,
                           family= binomial(link="logit"))


# option to prevent fitting models to different datasets
options(na.action = "na.fail")

# model selection
select.patsib.bin <- dredge(full.patsib.bin,
                            fixed = c("cond(four.region)", "cond(sex)",
                                      "cond(SES.class)"),
                            trace = 100, rank="AICc")
select.patsib.bin


# continuous part
# have to remove rows with missing mar1age

marriage.data.patsib.2 <- marriage.data.patsib %>%
  filter(!is.na(mar1age))

full.patsib.cont <- glmmTMB(mar1age ~ four.region + sex + SES.class +
                              father + father.sister + pat.cousins +
                              older.brothers + older.sisters +
                              younger.brothers + younger.sisters +
                              nephews + (1|mumid) + (1|cohort),
                            data = marriage.data.patsib.2,
                            family= gaussian(link="identity"))

# option to prevent fitting models to different datasets
options(na.action = "na.fail")

# model selection
select.patsib.cont <- dredge(full.patsib.cont,
                             fixed = c("cond(four.region)", "cond(sex)",
                                       "cond(SES.class)"),
                             trace = 100, rank="AICc")
select.patsib.cont
```

### **Model selection, maternal and siblings**

only those relatives who were selected in their respective models maternal: mother, maternal grandfather, mother’s sister sibling: nephews, all siblings

```
marriage.data.matsib <- child.data.final %>%
  dplyr::select(ID, evermarried, mar1age, mar1age.zi, sex, cohort, four.region,
                SES.class, mumid,mother, mother.father, mother.sister,
                older.brothers, older.sisters, younger.brothers,
                younger.sisters,nephews) %>%
  drop_na(!mar1age) %>%
  droplevels()

# select best-fitting model for maternal kin + siblings
# region, sex and SES class will be kept in all models
# maternal kin + siblings allowed to vary

# because of computational limitations, binomial and continuous part of the outcome run separately

full.matsib.bin <- glmmTMB(evermarried ~ four.region + sex + SES.class +
                             mother + mother.father + mother.sister +
                             older.brothers + older.sisters +
                             younger.brothers + younger.sisters +
                             nephews + (1|mumid) + (1|cohort),
                           data = marriage.data.matsib,
                           family= binomial(link="logit"))


# option to prevent fitting models to different datasets
options(na.action = "na.fail")

# model selection
select.matsib.bin <- dredge(full.matsib.bin,
                            fixed = c("cond(four.region)", "cond(sex)",
                                      "cond(SES.class)"),
                            trace = 100, rank="AICc")
select.matsib.bin


# continuous part
# have to remove rows with missing mar1age

marriage.data.matsib.2 <- marriage.data.matsib %>%
  filter(!is.na(mar1age))

full.matsib.cont <- glmmTMB(mar1age ~ four.region + sex + SES.class +
                              mother + mother.father + mother.sister +
                              older.brothers + older.sisters +
                              younger.brothers + younger.sisters +
                              nephews + (1|mumid) + (1|cohort),
                            data = marriage.data.matsib.2,
                            family= gaussian(link="identity"))

# option to prevent fitting models to different datasets
options(na.action = "na.fail")

# model selection
select.matsib.cont <- dredge(full.matsib.cont,
                             fixed = c("cond(four.region)", "cond(sex)",
                                       "cond(SES.class)"),
                             trace = 100, rank="AICc")
select.matsib.cont
```

### **Final model after model selection, paternal + siblings**

```
final.patsib <- glmmTMB(mar1age.zi ~ four.region + sex*SES.class*father +
                         sex*SES.class*older.brothers +
                          sex*SES.class*older.sisters +
                          sex*SES.class*younger.brothers +
                          sex*SES.class*younger.sisters +
                         sex*SES.class*nephews + (1|mumid) + (1|cohort),
                       ziformula = ~.,
                       dispformula = ~ 1,
                       data = marriage.data.patsib,
                       family= gaussian(link="identity"))
summary(final.patsib)

# anova
Anova(final.patsib,type="III")


# pairwise contrasts

# father
# binomial part
emms.father.bin <- cbind(pairs(emmeans(final.patsib, ~father*sex*SES.class,
                                       component="zi", type="response",
                                       rg.limit = 20000),adjust="none",reverse=T),
              confint(pairs(emmeans(final.patsib,  ~father*sex*SES.class, component="zi",type="response",rg.limit = 20000),adjust="none",reverse=T)))
emms.father.bin[c(1,6,15,28),c(1,6,7,9,12,13)]

# continuous part
emms.father.cont <- cbind(pairs(emmeans(final.patsib, ~father*sex*SES.class, component="cond", type="response",rg.limit = 20000),adjust="none",reverse=T),
              confint(pairs(emmeans(final.patsib,  ~father*sex*SES.class, component="cond",type="response",rg.limit = 20000),adjust="none",reverse=T)))
emms.father.cont[c(1,6,15,28),c(1,5,6,8,11,12)]


# older brothers
# binomial part
emms.oldbro.bin <- cbind(pairs(emmeans(final.patsib, ~older.brothers*sex*SES.class, component="zi", type="response",rg.limit = 20000),adjust="none",reverse=T),
                         confint(pairs(emmeans(final.patsib,
                                               ~older.brothers*sex*SES.class, component="zi",type="response",rg.limit = 20000),adjust="none",reverse=T)))
emms.oldbro.bin[c(1,2,10,14,28,35,55,65),c(1,6,7,9,12,13)]

# continuous part
emms.oldbro.cont <- cbind(pairs(emmeans(final.patsib, ~older.brothers*sex*SES.class, component="cond", type="response",rg.limit = 20000),adjust="none",reverse=T),
                          confint(pairs(emmeans(final.patsib,  ~older.brothers*sex*SES.class, component="cond",type="response",rg.limit = 20000),adjust="none",reverse=T)))
emms.oldbro.cont[c(1,2,10,14,28,35,55,65),c(1,5,6,8,11,12)]


# older sisters
# binomial part
emms.oldsis.bin <- cbind(pairs(emmeans(final.patsib, ~older.sisters*sex*SES.class, component="zi", type="response",rg.limit = 20000),adjust="none",reverse=T),
                         confint(pairs(emmeans(final.patsib,
                                               ~older.sisters*sex*SES.class, component="zi",type="response",rg.limit = 20000),adjust="none",reverse=T)))
emms.oldsis.bin[c(1,2,10,14,28,35,55,65),c(1,6,7,9,12,13)]

# continuous part
emms.oldsis.cont <- cbind(pairs(emmeans(final.patsib, ~older.sisters*sex*SES.class, component="cond", type="response",rg.limit = 20000),adjust="none",reverse=T),
                          confint(pairs(emmeans(final.patsib,
                                                ~older.sisters*sex*SES.class, component="cond",type="response",rg.limit = 20000),adjust="none",reverse=T)))
emms.oldsis.cont[c(1,2,10,14,28,35,55,65),c(1,5,6,8,11,12)]


# younger brothers
# binomial part
emms.youngbro.bin <- cbind(pairs(emmeans(final.patsib, ~younger.brothers*sex*SES.class, component="zi", type="response",rg.limit = 20000),adjust="none",reverse=T),
                         confint(pairs(emmeans(final.patsib,  ~younger.brothers*sex*SES.class, component="zi",type="response",rg.limit = 20000),adjust="none",reverse=T)))
emms.youngbro.bin[c(1,2,10,14,28,35,55,65),c(1,6,7,9,12,13)]

# continuous part
emms.youngbro.cont <- cbind(pairs(emmeans(final.patsib, ~younger.brothers*sex*SES.class, component="cond", type="response",rg.limit = 20000),adjust="none",reverse=T),
                          confint(pairs(emmeans(final.patsib,  ~younger.brothers*sex*SES.class, component="cond",type="response",rg.limit = 20000),adjust="none",reverse=T)))
emms.youngbro.cont[c(1,2,10,14,28,35,55,65),c(1,5,6,8,11,12)]


# younger sisters
# binomial part
emms.youngsis.bin <- cbind(pairs(emmeans(final.patsib, ~younger.sisters*sex*SES.class, component="zi", type="response",rg.limit = 20000),adjust="none",reverse=T),
                           confint(pairs(emmeans(final.patsib,  ~younger.sisters*sex*SES.class, component="zi",type="response",rg.limit = 20000),adjust="none",reverse=T)))
emms.youngsis.bin[c(1,2,10,14,28,35,55,65),c(1,6,7,9,12,13)]

# continuous part
emms.youngsis.cont <- cbind(pairs(emmeans(final.patsib, ~younger.sisters*sex*SES.class, component="cond", type="response",rg.limit = 20000),adjust="none",reverse=T),
                            confint(pairs(emmeans(final.patsib,  ~younger.sisters*sex*SES.class, component="cond",type="response",rg.limit = 20000),adjust="none",reverse=T)))
emms.youngsis.cont[c(1,2,10,14,28,35,55,65),c(1,5,6,8,11,12)]


# nephews
# binomial part
emms.neph.bin <- cbind(pairs(emmeans(final.patsib, ~nephews*sex*SES.class, component="zi", type="response",rg.limit = 20000),adjust="none",reverse=T),
                           confint(pairs(emmeans(final.patsib,  ~nephews*sex*SES.class, component="zi",type="response",rg.limit = 20000),adjust="none",reverse=T)))
emms.neph.bin[c(1,6,15,28),c(1,6,7,9,12,13)]

# continuous part
emms.neph.cont <- cbind(pairs(emmeans(final.patsib, ~nephews*sex*SES.class, component="cond", type="response",rg.limit = 20000),adjust="none",reverse=T),
                            confint(pairs(emmeans(final.patsib,  ~nephews*sex*SES.class, component="cond",type="response",rg.limit = 20000),adjust="none",reverse=T)))
emms.neph.cont[c(1,6,15,28),c(1,5,6,8,11,12)]
```

### **Final model after model selection, maternal + siblings**

older brothers, older sisters, younger brothers maternal grandfather, mother’s sister, nephews, older brothers, older sisters, younger brothers

```
final.matsib <- glmmTMB(mar1age.zi ~ four.region + sex*SES.class*mother.father +
                          sex*SES.class*mother.sister +
                          sex*SES.class*older.brothers +
                          sex*SES.class*older.sisters +
                          sex*SES.class*younger.brothers +
                          sex*SES.class*nephews + (1|mumid) + (1|cohort),
                        ziformula = ~.,
                        dispformula = ~ 1,
                        data = marriage.data.matsib,
                        family= gaussian(link="identity"))
summary(final.matsib)

# anova
Anova(final.matsib,type="III")


# pairwise contrasts

# maternal grandfather
# binomial part
emms.mgf.bin <- cbind(pairs(emmeans(final.matsib, ~mother.father*sex*SES.class, component="zi", type="response",rg.limit = 20000),adjust="none",reverse=T),
                         confint(pairs(emmeans(final.matsib,
                                               ~mother.father*sex*SES.class, component="zi",type="response",rg.limit = 20000),adjust="none",reverse=T)))
emms.mgf.bin[c(1,6,15,28),c(1,6,7,9,12,13)]

# continuous part
emms.mgf.cont <- cbind(pairs(emmeans(final.matsib, ~mother.father*sex*SES.class, component="cond", type="response",rg.limit = 20000),adjust="none",reverse=T),
                          confint(pairs(emmeans(final.matsib,
                                                ~mother.father*sex*SES.class, component="cond",type="response",rg.limit = 20000),adjust="none",reverse=T)))
emms.mgf.cont[c(1,6,15,28),c(1,5,6,8,11,12)]


# maternal aunt
# binomial part
emms.mataunt.bin <- cbind(pairs(emmeans(final.matsib, ~mother.sister*sex*SES.class, component="zi", type="response",rg.limit = 20000),adjust="none",reverse=T),
                      confint(pairs(emmeans(final.matsib,
                                            ~mother.sister*sex*SES.class, component="zi",type="response",rg.limit = 20000),adjust="none",reverse=T)))
emms.mataunt.bin[c(1,2,10,14,28,35,55,65),c(1,6,7,9,12,13)]

# continuous part
emms.mataunt.cont <- cbind(pairs(emmeans(final.matsib, ~mother.sister*sex*SES.class, component="cond", type="response",rg.limit = 20000),adjust="none",reverse=T),
                       confint(pairs(emmeans(final.matsib,
                                             ~mother.sister*sex*SES.class, component="cond",type="response",rg.limit = 20000),adjust="none",reverse=T)))
emms.mataunt.cont[c(1,2,10,14,28,35,55,65),c(1,5,6,8,11,12)]


# older brothers
# binomial part
emms.oldbro.bin <- cbind(pairs(emmeans(final.matsib, ~older.brothers*sex*SES.class, component="zi", type="response",rg.limit = 20000),adjust="none",reverse=T),
                         confint(pairs(emmeans(final.matsib,
                                               ~older.brothers*sex*SES.class, component="zi",type="response",rg.limit = 20000),adjust="none",reverse=T)))
emms.oldbro.bin[c(1,2,10,14,28,35,55,65),c(1,6,7,9,12,13)]

# continuous part
emms.oldbro.cont <- cbind(pairs(emmeans(final.matsib, ~older.brothers*sex*SES.class, component="cond", type="response",rg.limit = 20000),adjust="none",reverse=T),
                          confint(pairs(emmeans(final.matsib,  ~older.brothers*sex*SES.class, component="cond",type="response",rg.limit = 20000),adjust="none",reverse=T)))
emms.oldbro.cont[c(1,2,10,14,28,35,55,65),c(1,5,6,8,11,12)]


# older sisters
# binomial part
emms.oldsis.bin <- cbind(pairs(emmeans(final.matsib, ~older.sisters*sex*SES.class, component="zi", type="response",rg.limit = 20000),adjust="none",reverse=T),
                         confint(pairs(emmeans(final.matsib,  ~older.sisters*sex*SES.class, component="zi",type="response",rg.limit = 20000),adjust="none",reverse=T)))
emms.oldsis.bin[c(1,2,10,14,28,35,55,65),c(1,6,7,9,12,13)]

# continuous part
emms.oldsis.cont <- cbind(pairs(emmeans(final.matsib, ~older.sisters*sex*SES.class, component="cond", type="response",rg.limit = 20000),adjust="none",reverse=T),
                          confint(pairs(emmeans(final.matsib,  ~older.sisters*sex*SES.class, component="cond",type="response",rg.limit = 20000),adjust="none",reverse=T)))
emms.oldsis.cont[c(1,2,10,14,28,35,55,65),c(1,5,6,8,11,12)]


# younger brothers
# binomial part
emms.youngbro.bin <- cbind(pairs(emmeans(final.matsib, ~younger.brothers*sex*SES.class, component="zi", type="response",rg.limit = 20000),adjust="none",reverse=T),
                           confint(pairs(emmeans(final.matsib,  ~younger.brothers*sex*SES.class, component="zi",type="response",rg.limit = 20000),adjust="none",reverse=T)))
emms.youngbro.bin[c(1,2,10,14,28,35,55,65),c(1,6,7,9,12,13)]

# continuous part
emms.youngbro.cont <- cbind(pairs(emmeans(final.matsib, ~younger.brothers*sex*SES.class, component="cond", type="response",rg.limit = 20000),adjust="none",reverse=T),
                            confint(pairs(emmeans(final.matsib,  ~younger.brothers*sex*SES.class, component="cond",type="response",rg.limit = 20000),adjust="none",reverse=T)))
emms.youngbro.cont[c(1,2,10,14,28,35,55,65),c(1,5,6,8,11,12)]
# nephews
# binomial part
emms.neph.bin <- cbind(pairs(emmeans(final.matsib, ~nephews*sex*SES.class, component="zi", type="response",rg.limit = 20000),adjust="none",reverse=T),
                       confint(pairs(emmeans(final.matsib,  ~nephews*sex*SES.class, component="zi",type="response",rg.limit = 20000),adjust="none",reverse=T)))
emms.neph.bin[c(1,6,15,28),c(1,6,7,9,12,13)]

# continuous part
emms.neph.cont <- cbind(pairs(emmeans(final.matsib, ~nephews*sex*SES.class, component="cond", type="response",rg.limit = 20000),adjust="none",reverse=T),
                        confint(pairs(emmeans(final.matsib,  ~nephews*sex*SES.class, component="cond",type="response",rg.limit = 20000),adjust="none",reverse=T)))
emms.neph.cont[c(1,6,15,28),c(1,5,6,8,11,12)]
```

## **Lifetime number of children**

from the final data utilized in marriage, filter out 1) those with mar1age missing, 2) those that don’t have all kids known (have full life history and survived until 45)

categorize mar1age to be used as covariate add variable childless, where 1 = no children, 0 = at least 1 child

add variable children.number, from which childless removed

```
child.data.children <- child.data.final %>%
  filter(!is.na(mar1age)) %>%
  filter(allkidsknown == 1) %>%
  mutate(mar1age.class = case_when(mar1age < 20 ~ "under.20",
                                   mar1age >= 20 & mar1age < 25 ~ "20-25",
                                   mar1age >= 25 & mar1age < 30 ~ "25-30",
                                   mar1age >= 30 & mar1age < 35 ~ "30-35",
                                   mar1age >= 35 ~ "over.35",
                                   .default=NA),
         childless = case_when(kids == 0 ~ 1,
                               kids > 0 ~ 0,
                               .default = NA),
         children.number = case_when(kids == 0 ~ NA,
                                     kids > 0 ~ kids))

# order age at first marriage classes
child.data.children$mar1age.class <- factor(child.data.children$mar1age.class,
                                 levels=c("under.20","20-25","25-30",
                                          "30-35","over.35"))
```

### **Crosstabulation**

crosstabulation of childlessness and relatives separately for landless and landowner families

```
# landless
child.data.children.landless <- child.data.children %>%
  filter(SES.class == "landless")

table(child.data.children.landless$mother,child.data.children.landless$childless)
table(child.data.children.landless$father,child.data.children.landless$childless)
table(child.data.children.landless$father.mother,child.data.children.landless$childless)
table(child.data.children.landless$father.father,child.data.children.landless$childless) # only 9 that are childless and have pgf alive
table(child.data.children.landless$mother.mother,child.data.children.landless$childless)
table(child.data.children.landless$mother.father,child.data.children.landless$childless)

table(child.data.children.landless$older.brothers,child.data.children.landless$childless)
table(child.data.children.landless$older.sisters,child.data.children.landless$childless)
table(child.data.children.landless$younger.brothers,child.data.children.landless$childless)
table(child.data.children.landless$younger.sisters,child.data.children.landless$childless)

table(child.data.children.landless$father.sister,child.data.children.landless$childless)
table(child.data.children.landless$father.brother,child.data.children.landless$childless)
table(child.data.children.landless$mother.sister,child.data.children.landless$childless)
table(child.data.children.landless$mother.brother,child.data.children.landless$childless)

table(child.data.children.landless$pat.cousins,child.data.children.landless$childless) # only 4 that are childless and have 16 or more cousins
table(child.data.children.landless$mat.cousins,child.data.children.landless$childless) # only 6 that are childless and have 16 or more cousins

table(child.data.children.landless$nieces,child.data.children.landless$childless)
table(child.data.children.landless$nephews,child.data.children.landless$childless)


# landowner families
child.data.children.landed <- child.data.children %>%
  filter(SES.class == "landowner")

table(child.data.children.landed$mother,child.data.children.landed$childless)
table(child.data.children.landed$father,child.data.children.landed$childless)
table(child.data.children.landed$father.mother,child.data.children.landed$childless)
table(child.data.children.landed$father.father,child.data.children.landed$childless)
table(child.data.children.landed$mother.mother,child.data.children.landed$childless)
table(child.data.children.landed$mother.father,child.data.children.landed$childless)

table(child.data.children.landed$older.brothers,child.data.children.landed$childless)
table(child.data.children.landed$older.sisters,child.data.children.landed$childless)
table(child.data.children.landed$younger.brothers,child.data.children.landed$childless)
table(child.data.children.landed$younger.sisters,child.data.children.landed$childless)

table(child.data.children.landed$father.sister,child.data.children.landed$childless)
table(child.data.children.landed$father.brother,child.data.children.landed$childless)
table(child.data.children.landed$mother.sister,child.data.children.landed$childless)
table(child.data.children.landed$mother.brother,child.data.children.landed$childless)

table(child.data.children.landed$pat.cousins,child.data.children.landed$childless) # only 9 who are childless and have 16 or more cousins
table(child.data.children.landed$mat.cousins,child.data.children.landed$childless)

table(child.data.children.landed$nieces,child.data.children.landed$childless)
table(child.data.children.landed$nephews,child.data.children.landed$childless)


# sample size mostly enough for both
# if cousins are selected in model selection, have to group 6-10, 11-15 & 16+ classes together
```

### **Full model, paternal**

first create data with no missing information except in children.number

```
children.data.paternal <- child.data.children %>%
  dplyr::select(ID, kids, childless, children.number, sex, cohort, four.region, SES.class, mumid, mar1age.class,
                father, father.father, father.mother,
                father.brother, father.sister, pat.cousins) %>%
  drop_na(!children.number) %>%
  droplevels()
```

full model with region, sex and SES.class as fixed covariates paternal relatives also mumid, cohort and mar1age.class as random variables

```
# truncated poisson distribution
full.paternal <- glmmTMB(kids ~ four.region + sex + SES.class +
                           father + father.father + father.mother +
                           father.brother + father.sister + pat.cousins + (1|mumid) + (1|cohort) + (1|mar1age.class),
                         ziformula = ~.,
                         dispformula = ~ 1,
                         data = children.data.paternal,
                         family= truncated_poisson(link="log"))
```

model fit

```
sim.paternal <- simulateResiduals(fittedModel = full.paternal, plot = T)
# good residual plot
# good dispersion
testDispersion(sim.paternal)

# categorical variables
# all groups seem to have equal variance
testCategorical(sim.paternal, catPred = children.data.paternal$sex)
testCategorical(sim.paternal, catPred = children.data.paternal$four.region)
testCategorical(sim.paternal, catPred = children.data.paternal$SES.class)
testCategorical(sim.paternal, catPred = children.data.paternal$father)
testCategorical(sim.paternal, catPred = children.data.paternal$father.father)
testCategorical(sim.paternal, catPred = children.data.paternal$father.mother)
testCategorical(sim.paternal, catPred = children.data.paternal$father.brother)
testCategorical(sim.paternal, catPred = children.data.paternal$father.sister)
testCategorical(sim.paternal, catPred = children.data.paternal$pat.cousins)

# recalculate residuals by grouping by mumid
# this could show over or underdispersion
sim.paternal.2 <- recalculateResiduals(sim.paternal,
                                       group=children.data.paternal$mumid)
plot(sim.paternal.2)
# good dispersion
testDispersion(sim.paternal.2)


# compare to negbin distribution

full.paternal.negbin <- glmmTMB(kids ~ four.region + sex + SES.class +
                           father + father.father + father.mother +
                           father.brother + father.sister + pat.cousins +
                             (1|mumid) + (1|cohort) + (1|mar1age.class),
                         ziformula = ~.,
                         dispformula = ~ 1,
                         data = children.data.paternal,
                         family= truncated_nbinom2(link="log"))

# model fit

sim.paternal.negbin <- simulateResiduals(fittedModel = full.paternal.negbin,
                                         plot = T)
# good dispersion
testDispersion(sim.paternal.negbin)

# recalculate residuals by grouping by mumid
# this could show over or underdispersion
sim.paternal.negbin.2 <- recalculateResiduals(sim.paternal.negbin,
                                              group=children.data.paternal$mumid)
plot(sim.paternal.negbin.2)
# good dispersion
testDispersion(sim.paternal.negbin.2)


# both models look good, let's use poisson distribution
```

multicollinearity

check_collinearity(full.paternal, component = c('all'))

### **Model selection, paternal**

select best-fitting model for paternal kin region, sex and SES class will be kept in all models paternal kin allowed to vary

because of computational limitations, binomial and continuous part of the outcome run separately

```
# binomial part
full.paternal.bin <- glmmTMB(childless ~ four.region + sex + SES.class +
                               father + father.father + father.mother +
                               father.brother + father.sister + pat.cousins +
                               (1|mumid) + (1|cohort) + (1|mar1age.class),
                             data = children.data.paternal,
                             family= binomial(link="logit"))

# option to prevent fitting models to different datasets
options(na.action = "na.fail")

# model selection
select.paternal.bin <- dredge(full.paternal.bin,
                              fixed = c("cond(four.region)", "cond(sex)",
                                        "cond(SES.class)"),
                              trace = 100, rank="AICc")
select.paternal.bin


# numerical part
# have to remove rows with missing children.number

children.data.paternal.2 <- children.data.paternal %>%
  filter(!is.na(children.number))

full.paternal.cont <- glmmTMB(children.number ~ four.region + sex + SES.class +
                                father + father.father + father.mother +
                                father.brother + father.sister + pat.cousins +
                                (1|mumid) + (1|cohort) + (1|mar1age.class),
                              data = children.data.paternal.2,
                              family= poisson(link="log"))

# option to prevent fitting models to different datasets
options(na.action = "na.fail")

# model selection
select.paternal.cont <- dredge(full.paternal.cont,
                               fixed = c("cond(four.region)", "cond(sex)",
                                         "cond(SES.class)"),
                               trace = 100, rank="AICc")
select.paternal.cont
```

### **Full model, maternal**

first create data with no missing information except in children.number

```
children.data.maternal <- child.data.children %>%
  dplyr::select(ID, kids, childless, children.number, sex, cohort, four.region, SES.class, mumid, mar1age.class,
                mother, mother.father, mother.mother,
                mother.brother, mother.sister, mat.cousins) %>%
  drop_na(!children.number) %>%
  droplevels()
```

full model with region, sex and SES.class as fixed covariates maternal relatives also mumid, cohort and mar1age.class as random variables

```
# truncated poisson distribution
full.maternal <- glmmTMB(kids ~ four.region + sex + SES.class +
                           mother + mother.father + mother.mother +
                           mother.brother + mother.sister + mat.cousins + (1|mumid) + (1|cohort) + (1|mar1age.class),
                         ziformula = ~.,
                         dispformula = ~ 1,
                         data = children.data.maternal,
                         family= truncated_poisson(link="log"))
```

model fit

```
sim.maternal <- simulateResiduals(fittedModel = full.maternal, plot = T)
# good residual plot
# good dispersion
testDispersion(sim.maternal)

# categorical variables
# all groups seem to have equal variance
testCategorical(sim.maternal, catPred = children.data.maternal$sex)
testCategorical(sim.maternal, catPred = children.data.maternal$four.region)
testCategorical(sim.maternal, catPred = children.data.maternal$SES.class)
testCategorical(sim.maternal, catPred = children.data.maternal$mother)
testCategorical(sim.maternal, catPred = children.data.maternal$mother.father)
testCategorical(sim.maternal, catPred = children.data.maternal$mother.mother)
testCategorical(sim.maternal, catPred = children.data.maternal$mother.brother)
testCategorical(sim.maternal, catPred = children.data.maternal$mother.sister)
testCategorical(sim.maternal, catPred = children.data.maternal$mat.cousins)


# recalculate residuals by grouping by mumid
# this could show over or underdispersion
sim.maternal.2 <- recalculateResiduals(sim.maternal,
                                       group=children.data.maternal$mumid)
plot(sim.maternal.2)
# good dispersion
testDispersion(sim.maternal.2)


# compare to negbin distribution

full.maternal.negbin <- glmmTMB(kids ~ four.region + sex + SES.class +
                                  mother + mother.father + mother.mother +
                                  mother.brother + mother.sister + mat.cousins +
                                  (1|mumid) + (1|cohort) + (1|mar1age.class),
                                ziformula = ~.,
                                dispformula = ~ 1,
                                data = children.data.maternal,
                                family= truncated_nbinom2(link="log"))

# model fit

sim.maternal.negbin <- simulateResiduals(fittedModel = full.maternal.negbin,
                                         plot = T)
# good dispersion
testDispersion(sim.maternal.negbin)

# recalculate residuals by grouping by mumid
# this could show over or underdispersion
sim.maternal.negbin.2 <- recalculateResiduals(sim.maternal.negbin,
                                              group=children.data.maternal$mumid)
plot(sim.maternal.negbin.2)
# good dispersion
testDispersion(sim.maternal.negbin.2)

# both models look good, let's use poisson distribution
```

multicollinearity

check_collinearity(full.maternal, component = c('all'))

### **Model selection, maternal**

select best-fitting model for maternal kin region, sex and SES class will be kept in all models maternal kin allowed to vary

because of computational limitations, binomial and continuous part of the outcome run separately

```
#binomial part
full.maternal.bin <- glmmTMB(childless ~ four.region + sex + SES.class +
                               mother + mother.father + mother.mother +
                               mother.brother + mother.sister + mat.cousins +
                               (1|mumid) + (1|cohort) + (1|mar1age.class),
                             data = children.data.maternal,
                             family= binomial(link="logit"))

# option to prevent fitting models to different datasets
options(na.action = "na.fail")

# model selection
select.maternal.bin <- dredge(full.maternal.bin,
                              fixed = c("cond(four.region)", "cond(sex)",
                                        "cond(SES.class)"),
                              trace = 100, rank="AICc")
select.maternal.bin


# numerical part
# have to remove rows with missing children.number

children.data.maternal.2 <- children.data.maternal %>%
  filter(!is.na(children.number))

full.maternal.cont <- glmmTMB(children.number ~ four.region + sex + SES.class +
                                mother + mother.father + mother.mother +
                                mother.brother + mother.sister + mat.cousins +
                                (1|mumid) + (1|cohort) + (1|mar1age.class),
                              data = children.data.maternal.2,
                              family= poisson(link="log"))

# option to prevent fitting models to different datasets
options(na.action = "na.fail")

# model selection
select.maternal.cont <- dredge(full.maternal.cont,
                               fixed = c("cond(four.region)", "cond(sex)",
                                         "cond(SES.class)"),
                               trace = 100, rank="AICc")
select.maternal.cont
```

### **Full model, siblings**

first create data with no missing information except in children.number

```
children.data.sibnib <- child.data.children %>%
  dplyr::select(ID, kids, childless, children.number, sex, cohort, four.region,
                SES.class, mumid, mar1age.class, older.brothers, older.sisters,
                younger.brothers, younger.sisters, nephews, nieces) %>%
  drop_na(!children.number) %>%
  droplevels()
```

full model with region, sex and SES.class as fixed covariates siblings + niblings also mumid, cohort and mar1age.class as random variables

```
# truncated poisson distribution
full.sibnib <- glmmTMB(kids ~ four.region + sex + SES.class +
                         older.brothers + older.sisters + younger.brothers +
                         younger.sisters + nephews + nieces +
                         (1|mumid) + (1|cohort) + (1|mar1age.class),
                         ziformula = ~.,
                         dispformula = ~ 1,
                         data = children.data.sibnib,
                         family= truncated_poisson(link="log"))
```

model fit

```
sim.sibnib <- simulateResiduals(fittedModel = full.sibnib, plot = T)
# good residual plot
# good dispersion
testDispersion(sim.sibnib)

# categorical variables
# all groups seem to have equal variance
testCategorical(sim.sibnib, catPred = children.data.sibnib$sex)
testCategorical(sim.sibnib, catPred = children.data.sibnib$four.region)
testCategorical(sim.sibnib, catPred = children.data.sibnib$SES.class)
testCategorical(sim.sibnib, catPred = children.data.sibnib$older.brothers)
testCategorical(sim.sibnib, catPred = children.data.sibnib$older.sisters)
testCategorical(sim.sibnib, catPred = children.data.sibnib$younger.brothers)
testCategorical(sim.sibnib, catPred = children.data.sibnib$younger.sisters)
testCategorical(sim.sibnib, catPred = children.data.sibnib$nephews)
testCategorical(sim.sibnib, catPred = children.data.sibnib$nieces)

# recalculate residuals by grouping by mumid
# this could show over or underdispersion
sim.sibnib.2 <- recalculateResiduals(sim.sibnib,
                                     group=children.data.sibnib$mumid)
plot(sim.sibnib.2)
# good dispersion
testDispersion(sim.sibnib.2)


# compare to negbin distribution

full.sibnib.negbin <- glmmTMB(kids ~ four.region + sex + SES.class +
                                older.brothers + older.sisters +
                                younger.brothers + younger.sisters +
                                nephews + nieces + (1|mumid) + (1|cohort) +
                                (1|mar1age.class),
                                ziformula = ~.,
                                dispformula = ~ 1,
                                data = children.data.sibnib,
                                family= truncated_nbinom2(link="log"))

# model fit

sim.sibnib.negbin <- simulateResiduals(fittedModel = full.sibnib.negbin,
                                       plot = T)
# good dispersion
testDispersion(sim.sibnib.negbin)

# recalculate residuals by grouping by mumid
# this could show over or underdispersion
sim.sibnib.negbin.2 <- recalculateResiduals(sim.sibnib.negbin,
                                            group=children.data.sibnib$mumid)
plot(sim.sibnib.negbin.2)
# good dispersion
testDispersion(sim.sibnib.negbin.2)

# both models look good, let's use poisson distribution
```

multicollinearity

check_collinearity(full.sibnib, component = c('all'))

### **Model selection, siblings**

select best-fitting model for siblings and niblings region, sex and SES class will be kept in all models siblings and niblings allowed to vary

because of computational limitations, binomial and continuous part of the outcome run separately

```
# binomial part
full.sibnib.bin <- glmmTMB(childless ~ four.region + sex + SES.class +
                             older.brothers + older.sisters + younger.brothers +
                             younger.sisters + nephews + nieces +
                             (1|mumid) + (1|cohort) + (1|mar1age.class),
                             data = children.data.sibnib,
                             family = binomial(link="logit"))

# option to prevent fitting models to different datasets
options(na.action = "na.fail")

# model selection
select.sibnib.bin <- dredge(full.sibnib.bin,
                            fixed = c("cond(four.region)", "cond(sex)",
                                      "cond(SES.class)"),
                              trace = 100, rank="AICc")
select.sibnib.bin


# numerical part
# have to remove rows with missing children.number
children.data.sibnib.2 <- children.data.sibnib %>%
  filter(!is.na(children.number))

full.sibnib.cont <- glmmTMB(children.number ~ four.region + sex + SES.class +
                              older.brothers + older.sisters +
                              younger.brothers + younger.sisters +
                              nephews + nieces +
                              (1|mumid) + (1|cohort) + (1|mar1age.class),
                              data = children.data.sibnib.2,
                              family= poisson(link="log"))

# option to prevent fitting models to different datasets
options(na.action = "na.fail")

# model selection
select.sibnib.cont <- dredge(full.sibnib.cont,
                             fixed = c("cond(four.region)", "cond(sex)",
                                       "cond(SES.class)"),
                               trace = 100, rank="AICc")
select.sibnib.cont
```

### **Model selection, paternal and siblings**

only kin that were selected in the previous model selection

first create data with no missing information except in children.number

have to group paternal cousins further, as have too few observations for childless individuals

```
children.data.patsib <- child.data.children %>%
  dplyr::select(ID, kids, childless, children.number, sex, cohort, four.region,
                SES.class, mumid, mar1age.class, pat.cousins, father.brother,
                younger.brothers, younger.sisters,nieces) %>%
  drop_na(!children.number) %>%
  droplevels() %>%
  mutate(pat.cousins.comb = case_when(pat.cousins == "6-10" ~ "6 or more",
                                      pat.cousins == "11-15" ~ "6 or more",
                                      pat.cousins == "16 or more" ~ "6 or more",
                                      .default = pat.cousins),
         pat.cousins.comb = as.factor(pat.cousins.comb))

# order cousin factor levels
children.data.patsib$pat.cousins.comb <- factor(children.data.patsib$pat.cousins.comb,
                                        levels=c("0","1-5","6 or more"))
```

select best-fitting model for paternal kin + siblings region, sex and SES class will be kept in all models paternal kin + siblings allowed to vary

because of computational limitations, binomial and continuous part of the outcome run separately

```
# binomial part
full.patsib.bin <- glmmTMB(childless ~ four.region + sex + SES.class +
                             pat.cousins.comb + father.brother +
                             younger.brothers + younger.sisters + nieces +
                             (1|mumid) + (1|cohort) + (1|mar1age.class),
                           data = children.data.patsib,
                           family = binomial(link="logit"))

# option to prevent fitting models to different datasets
options(na.action = "na.fail")

# model selection
select.patsib.bin <- dredge(full.patsib.bin,
                            fixed = c("cond(four.region)", "cond(sex)",
                                      "cond(SES.class)"),
                            trace = 100, rank="AICc")
select.patsib.bin


# numerical part
# have to remove rows with missing children.number
children.data.patsib.2 <- children.data.patsib %>%
  filter(!is.na(children.number))

full.patsib.cont <- glmmTMB(children.number ~ four.region + sex + SES.class +
                              pat.cousins.comb + father.brother +
                              younger.brothers + younger.sisters + nieces +
                              (1|mumid) + (1|cohort) + (1|mar1age.class),
                              data = children.data.patsib.2,
                              family= poisson(link="log"))

# option to prevent fitting models to different datasets
options(na.action = "na.fail")

# model selection
select.patsib.cont <- dredge(full.patsib.cont,
                             fixed = c("cond(four.region)", "cond(sex)",
                                       "cond(SES.class)"),
                            trace = 100, rank="AICc")
select.patsib.cont
```

### **Model selection, maternal and siblings**

only kin that were selected in the previous model selection

first create data with no missing information except in children.number

have to group paternal cousins further, as have too few observations for childless individuals

```
children.data.matsib <- child.data.children %>%
  dplyr::select(ID, kids, childless, children.number, sex, cohort, four.region,
                SES.class, mumid, mar1age.class, mat.cousins, mother.father,
                mother.mother, younger.brothers, younger.sisters,nieces) %>%
  drop_na(!children.number) %>%
  droplevels() %>%
  mutate(mat.cousins.comb = case_when(mat.cousins == "6-10" ~ "6 or more",
                                      mat.cousins == "11-15" ~ "6 or more",
                                      mat.cousins == "16 or more" ~ "6 or more",
                                      .default = mat.cousins),
         mat.cousins.comb = as.factor(mat.cousins.comb))

# order maternal cousin levels
children.data.matsib$mat.cousins.comb <- factor(children.data.matsib$mat.cousins.comb,
                                                levels=c("0","1-5","6 or more"))
```

select best-fitting model for maternal kin + siblings region, sex and SES class will be kept in all models maternal kin + siblings allowed to vary

because of computational limitations, binomial and continuous part of the outcome run separately

```
# binomial part
full.matsib.bin <- glmmTMB(childless ~ four.region + sex + SES.class +
                             mat.cousins.comb + mother.father + mother.mother +
                             younger.brothers + younger.sisters + nieces +
                             (1|mumid) + (1|cohort) + (1|mar1age.class),
                           data = children.data.matsib,
                           family = binomial(link="logit"))

# option to prevent fitting models to different datasets
options(na.action = "na.fail")

# model selection
select.matsib.bin <- dredge(full.matsib.bin,
                            fixed = c("cond(four.region)", "cond(sex)",
                                      "cond(SES.class)"),
                            trace = 100, rank="AICc")
select.matsib.bin


# numerical part
# have to remove rows with missing children.number
children.data.matsib.2 <- children.data.matsib %>%
  filter(!is.na(children.number))

full.matsib.cont <- glmmTMB(children.number ~ four.region + sex + SES.class +
                              mat.cousins.comb + mother.father + mother.mother +
                              younger.brothers + younger.sisters + nieces +
                              (1|mumid) + (1|cohort) + (1|mar1age.class),
                            data = children.data.matsib.2,
                            family= poisson(link="log"))

# option to prevent fitting models to different datasets
options(na.action = "na.fail")

# model selection
select.matsib.cont <- dredge(full.matsib.cont,
                             fixed = c("cond(four.region)", "cond(sex)",
                                       "cond(SES.class)"),
                             trace = 100, rank="AICc")
select.matsib.cont
```

## **Final models**

### **Marriage, siblings**

as neither paternal or maternal kin were significantly associated with either of the outcomes, only siblings retained in the final model to increase sample size and avoid fitting two models with exactly the same relatives but different sample

```
final.sib <- glmmTMB(mar1age.zi ~ four.region + sex*SES.class*older.brothers +
                       sex*SES.class*older.sisters +
                       sex*SES.class*younger.brothers +
                       sex*SES.class*younger.sisters + sex*SES.class*nephews +
                       (1|mumid) + (1|cohort),
                        ziformula = ~.,
                        dispformula = ~ 1,
                        data = marriage.data.sibnib,
                        family= gaussian(link="identity"))
summary(final.sib)

# anova
Anova(final.sib,type="III")

# pairwise contrasts


# older brothers
# binomial part
emms.oldbro.bin <- cbind(pairs(emmeans(final.sib, ~older.brothers*sex*SES.class,
                                       component="zi", type="response",
                                       rg.limit = 20000),adjust="none",
                               reverse=T),
                         confint(pairs(emmeans(final.sib,
                                               ~older.brothers*sex*SES.class,
                                               component="zi",type="response",
                                               rg.limit = 20000),adjust="none",
                                       reverse=T)))
emms.oldbro.bin[c(1,2,10,14,28,35,55,65),c(1,6,7,9,12,13)]

# continuous part
emms.oldbro.cont <- cbind(pairs(emmeans(final.sib, ~older.brothers*sex*SES.class,
                                        component="cond", type="response",
                                        rg.limit = 20000),adjust="none",
                                reverse=T),
                          confint(pairs(emmeans(final.sib,
                                                ~older.brothers*sex*SES.class,
                                                component="cond",type="response",
                                                rg.limit = 20000),adjust="none",
                                        reverse=T)))
emms.oldbro.cont[c(1,2,10,14,28,35,55,65),c(1,5,6,8,11,12)]


# older sisters
# binomial part
emms.oldsis.bin <- cbind(pairs(emmeans(final.sib, ~older.sisters*sex*SES.class,
                                       component="zi", type="response",
                                       rg.limit = 20000),adjust="none",
                               reverse=T),
                         confint(pairs(emmeans(final.sib,
                                               ~older.sisters*sex*SES.class,
                                               component="zi",type="response",
                                               rg.limit = 20000),adjust="none",
                                       reverse=T)))
emms.oldsis.bin[c(1,2,10,14,28,35,55,65),c(1,6,7,9,12,13)]

# continuous part
emms.oldsis.cont <- cbind(pairs(emmeans(final.sib, ~older.sisters*sex*SES.class,
                                        component="cond", type="response",
                                        rg.limit = 20000),adjust="none",
                                reverse=T),
                          confint(pairs(emmeans(final.sib,
                                                ~older.sisters*sex*SES.class,
                                                component="cond",type="response",
                                                rg.limit = 20000),adjust="none",
                                        reverse=T)))
emms.oldsis.cont[c(1,2,10,14,28,35,55,65),c(1,5,6,8,11,12)]


# younger brothers
# binomial part
emms.youngbro.bin <- cbind(pairs(emmeans(final.sib, ~younger.brothers*sex*SES.class,
                                         component="zi", type="response",
                                         rg.limit = 20000),adjust="none",reverse=T),
                           confint(pairs(emmeans(final.sib,
                                                 ~younger.brothers*sex*SES.class,
                                                 component="zi",type="response",
                                                 rg.limit = 20000),adjust="none",
                                         reverse=T)))
emms.youngbro.bin[c(1,2,10,14,28,35,55,65),c(1,6,7,9,12,13)]

# continuous part
emms.youngbro.cont <- cbind(pairs(emmeans(final.sib, ~younger.brothers*sex*SES.class,
                                          component="cond", type="response",
                                          rg.limit = 20000),adjust="none",reverse=T),
                            confint(pairs(emmeans(final.sib,
                                                  ~younger.brothers*sex*SES.class,
                                                  component="cond",type="response",
                                                  rg.limit = 20000),adjust="none",
                                          reverse=T)))
emms.youngbro.cont[c(1,2,10,14,28,35,55,65),c(1,5,6,8,11,12)]


# younger sisters
# binomial part
emms.youngsis.bin <- cbind(pairs(emmeans(final.sib, ~younger.sisters*sex*SES.class,
                                         component="zi", type="response",
                                         rg.limit = 20000),adjust="none",reverse=T),
                           confint(pairs(emmeans(final.sib,
                                                 ~younger.sisters*sex*SES.class,
                                                 component="zi",type="response",
                                                 rg.limit = 20000),adjust="none",
                                         reverse=T)))
emms.youngsis.bin[c(1,2,10,14,28,35,55,65),c(1,6,7,9,12,13)]

# continuous part
emms.youngsis.cont <- cbind(pairs(emmeans(final.sib, ~younger.sisters*sex*SES.class,
                                          component="cond", type="response",
                                          rg.limit = 20000),adjust="none",reverse=T),
                            confint(pairs(emmeans(final.sib,
                                                  ~younger.sisters*sex*SES.class,
                                                  component="cond",type="response",
                                                  rg.limit = 20000),adjust="none",
                                          reverse=T)))
emms.youngsis.cont[c(1,2,10,14,28,35,55,65),c(1,5,6,8,11,12)]


# nephews
# binomial part
emms.neph.bin <- cbind(pairs(emmeans(final.sib, ~nephews*sex*SES.class,
                                     component="zi", type="response",
                                     rg.limit = 20000),adjust="none",reverse=T),
                       confint(pairs(emmeans(final.sib,  ~nephews*sex*SES.class,
                                             component="zi",type="response",
                                             rg.limit = 20000),adjust="none",
                                     reverse=T)))
emms.neph.bin[c(1,6,15,28),c(1,6,7,9,12,13)]

# continuous part
emms.neph.cont <- cbind(pairs(emmeans(final.sib, ~nephews*sex*SES.class,
                                      component="cond", type="response",
                                      rg.limit = 20000),adjust="none",
                              reverse=T),
                        confint(pairs(emmeans(final.sib,
                                              ~nephews*sex*SES.class,
                                              component="cond",type="response",
                                              rg.limit = 20000),adjust="none",
                                      reverse=T)))
emms.neph.cont[c(1,6,15,28),c(1,5,6,8,11,12)]
```

### **Children, paternal and siblings**

```
final.patsib <- glmmTMB(kids ~ four.region + sex + SES.class +
                          sex*SES.class*father.brother +
                          sex*SES.class*pat.cousins.comb +
                          sex*SES.class*younger.brothers +
                          sex*SES.class*younger.sisters +
                         (1|mumid) + (1|cohort) + (1|mar1age.class),
                       ziformula = ~.,
                       dispformula = ~ 1,
                       data = children.data.patsib,
                       family= truncated_poisson(link="log"))

summary(final.patsib)

# anova
Anova(final.patsib,type="III")


# pairwise contrasts

# transformation to get percentage differences in number of children, instead of meaningless ratios
# based on https://stats.stackexchange.com/questions/551695/how-to-calculate-percentage-difference-of-geometric-means-with-emmeans and https://cran.r-project.org/web/packages/emmeans/vignettes/transformations.html#altscale
mytran = list(
  linkfun = function(mu) log(mu/100 + 1),
  linkinv = function(eta) 100 * (exp(eta) - 1),
  mu.eta = function(eta) 100 * exp(eta),
  name = "pct.diff tran")
# increase number of digits
emm_options(opt.digits = FALSE)


# uncles
# binomial part
emms.uncle.bin <- cbind(pairs(emmeans(final.patsib, ~father.brother*sex*SES.class,
                                      component="zi", type="response",
                                      rg.limit = 20000),adjust="none",reverse=T),
                         confint(pairs(emmeans(final.patsib,
                                               ~father.brother*sex*SES.class,
                                               component="zi",type="response",
                                               rg.limit = 20000),adjust="none",
                                       reverse=T)))
emms.uncle.bin[c(1,2,10,14,28,35,55,65),c(1,6,7,9,12,13)]

# continuous part
emms.uncle.cont <- pairs(emmeans(final.patsib, ~father.brother*sex*SES.class,
                                 rg.limit = 20000, component="cond",
                                 type="response"),adjust="none",reverse=T)
update(emms.uncle.cont, tran = mytran, inv.lbl = "pct.diff", infer = TRUE)


# paternal cousins
# binomial part
emms.cousin.bin <- cbind(pairs(emmeans(final.patsib, ~pat.cousins.comb*sex*SES.class,
                                       component="zi", type="response"
                                       ,rg.limit = 20000),adjust="none",reverse=T),
                        confint(pairs(emmeans(final.patsib,
                                              ~pat.cousins.comb*sex*SES.class,
                                              component="zi",type="response",
                                              rg.limit = 20000),adjust="none",
                                      reverse=T)))
emms.cousin.bin[c(1,2,10,14,28,35,55,65),c(1,6,7,9,12,13)]

# continuous part
emms.cousin.cont <- pairs(emmeans(final.patsib, ~pat.cousins.comb*sex*SES.class,
                                  rg.limit = 20000, component="cond",
                                  type="response"),adjust="none",reverse=T)
update(emms.cousin.cont, tran = mytran, inv.lbl = "pct.diff", infer = TRUE)


# younger brothers
# binomial part
emms.youngbro.bin <- cbind(pairs(emmeans(final.patsib, ~younger.brothers*sex*SES.class,
                                         component="zi", type="response",
                                         rg.limit = 20000),adjust="none",reverse=T),
                         confint(pairs(emmeans(final.patsib,
                                               ~younger.brothers*sex*SES.class,
                                               component="zi",type="response",
                                               rg.limit = 20000),adjust="none",
                                       reverse=T)))
emms.youngbro.bin[c(1,2,10,14,28,35,55,65),c(1,6,7,9,12,13)]

# continuous part
emms.youngbro.cont <- pairs(emmeans(final.patsib, ~younger.brothers*sex*SES.class,
                                    rg.limit = 20000, component="cond",
                                    type="response"),adjust="none",reverse=T)
update(emms.youngbro.cont, tran = mytran, inv.lbl = "pct.diff", infer = TRUE)


# younger sisters
# binomial part
emms.youngsis.bin <- cbind(pairs(emmeans(final.patsib, ~younger.sisters*sex*SES.class,
                                         component="zi", type="response",
                                         rg.limit = 20000),adjust="none",reverse=T),
                           confint(pairs(emmeans(final.patsib,
                                                 ~younger.sisters*sex*SES.class,
                                                 component="zi",type="response",
                                                 rg.limit = 20000),adjust="none",
                                         reverse=T)))
emms.youngsis.bin[c(1,2,10,14,28,35,55,65),c(1,6,7,9,12,13)]

# continuous part
emms.youngsis.cont <- pairs(emmeans(final.patsib, ~younger.sisters*sex*SES.class,
                                    rg.limit = 20000, component="cond",
                                    type="response"),adjust="none",reverse=T)
update(emms.youngsis.cont, tran = mytran, inv.lbl = "pct.diff", infer = TRUE)
```

### **Children, maternal and siblings**

```
final.matsib <- glmmTMB(kids ~ four.region + sex + SES.class +
                          sex*SES.class*mother.mother +
                          sex*SES.class*mat.cousins.comb +
                          sex*SES.class*younger.brothers +
                          sex*SES.class*younger.sisters +
                          sex*SES.class*nieces +
                          (1|mumid) + (1|cohort) + (1|mar1age.class),
                        ziformula = ~.,
                        dispformula = ~ 1,
                        data = children.data.matsib,
                        family= truncated_poisson(link="log"))

summary(final.matsib)

# anova
Anova(final.matsib,type="III")


# pairwise contrasts

# maternal grandmother
# binomial part
emms.mgm.bin <- cbind(pairs(emmeans(final.matsib, ~mother.mother*sex*SES.class,
                                    component="zi", type="response",
                                    rg.limit = 20000),adjust="none",reverse=T),
                      confint(pairs(emmeans(final.matsib,
                                            ~mother.mother*sex*SES.class,
                                            component="zi",type="response",
                                            rg.limit = 20000),adjust="none",
                                    reverse=T)))
emms.mgm.bin[c(1,6,15,28),c(1,6,7,9,12,13)]

# continuous part
emms.mgm.cont <- pairs(emmeans(final.matsib, ~mother.mother*sex*SES.class,
                               rg.limit = 20000, component="cond",
                               type="response"),adjust="none",reverse=T)
update(emms.mgm.cont, tran = mytran, inv.lbl = "pct.diff", infer = TRUE)


# maternal cousins
# binomial part
emms.cousin.bin <- cbind(pairs(emmeans(final.matsib, ~mat.cousins.comb*sex*SES.class,
                                       component="zi", type="response",
                                       rg.limit = 20000),adjust="none",reverse=T),
                         confint(pairs(emmeans(final.matsib,
                                               ~mat.cousins.comb*sex*SES.class,
                                               component="zi",type="response",
                                               rg.limit = 20000),adjust="none",
                                       reverse=T)))
emms.cousin.bin[c(1,2,10,14,28,35,55,65),c(1,6,7,9,12,13)]
# continuous part
emms.cousin.cont <- pairs(emmeans(final.matsib, ~mat.cousins.comb*sex*SES.class,
                                  rg.limit = 20000, component="cond",
                                  type="response"),adjust="none",reverse=T)
update(emms.cousin.cont, tran = mytran, inv.lbl = "pct.diff", infer = TRUE)


# younger brothers
# binomial part
emms.youngbro.bin <- cbind(pairs(emmeans(final.matsib, ~younger.brothers*sex*SES.class,
                                         component="zi", type="response",
                                         rg.limit = 20000),adjust="none",reverse=T),
                           confint(pairs(emmeans(final.matsib,
                                                 ~younger.brothers*sex*SES.class,
                                                 component="zi",type="response",
                                                 rg.limit = 20000),adjust="none",
                                         reverse=T)))
emms.youngbro.bin[c(1,2,10,14,28,35,55,65),c(1,6,7,9,12,13)]

# continuous part
emms.youngbro.cont <- pairs(emmeans(final.matsib, ~younger.brothers*sex*SES.class,
                                    rg.limit = 20000, component="cond",
                                    type="response"),adjust="none",reverse=T)
update(emms.youngbro.cont, tran = mytran, inv.lbl = "pct.diff", infer = TRUE)


# younger sisters
# binomial part
emms.youngsis.bin <- cbind(pairs(emmeans(final.matsib, ~younger.sisters*sex*SES.class,
                                         component="zi", type="response",
                                         rg.limit = 20000),adjust="none",reverse=T),
                           confint(pairs(emmeans(final.matsib,  ~younger.sisters*sex*SES.class, component="zi",type="response",rg.limit = 20000),adjust="none",reverse=T)))
emms.youngsis.bin[c(1,2,10,14,28,35,55,65),c(1,6,7,9,12,13)]

# continuous part
emms.youngsis.cont <- pairs(emmeans(final.matsib, ~younger.sisters*sex*SES.class,
                                    rg.limit = 20000, component="cond",
                                    type="response"),adjust="none",reverse=T)
update(emms.youngsis.cont, tran = mytran, inv.lbl = "pct.diff", infer = TRUE)


# nieces
# binomial part
emms.niece.bin <- cbind(pairs(emmeans(final.matsib, ~nieces*sex*SES.class,
                                      component="zi", type="response",
                                      rg.limit = 20000),adjust="none",
                              reverse=T),
                      confint(pairs(emmeans(final.matsib,  ~nieces*sex*SES.class,
                                            component="zi",type="response",
                                            rg.limit = 20000),adjust="none",
                                    reverse=T)))
emms.niece.bin[c(1,6,15,28),c(1,6,7,9,12,13)]

# continuous part
emms.niece.cont <- pairs(emmeans(final.matsib, ~nieces*sex*SES.class,
                                 rg.limit = 20000, component="cond",
                                 type="response"),adjust="none",reverse=T)
update(emms.niece.cont, tran = mytran, inv.lbl = "pct.diff", infer = TRUE)
```

## **Descriptive results**

% marrying

```
proportions(table(married=marriage.data.sibnib$evermarried,
                  sex=marriage.data.sibnib$sex,
                  SES=marriage.data.sibnib$SES.class),c("sex","SES"))
```

mean age at first marriage

```
marriage.data.sibnib %>%
  group_by(sex,SES.class) %>%
  summarize(meanage = mean(mar1age,na.rm=T))
```

% overlap in children datasets

```
# simplify datasets
children.data.patsib.s <- children.data.patsib %>%
  dplyr::select(ID,children.number,childless,sex,SES.class) %>%
  mutate(source="paternal")

children.data.matsib.s <- children.data.matsib %>%
  dplyr::select(ID,children.number,childless,sex,SES.class) %>%
  mutate(source="maternal")

# bind rows
# count IDs that appear in both datasets

children.data.comb <- rbind(children.data.patsib.s,children.data.matsib.s)
children.data.comb <- children.data.comb %>%
  group_by(ID) %>%
  add_count() %>%
  distinct(ID,.keep_all = T)

# % overlap in table
table(overlap=children.data.comb$n,source=children.data.comb$source)
```

% childless

```
proportions(table(childless=children.data.comb$childless,
                  sex=children.data.comb$sex,
                  SES=children.data.comb$SES.class),c("sex","SES"))
```

mean number of children

```
children.data.comb %>%
  group_by(sex,SES.class) %>%
  summarize(meankids = mean(children.number,na.rm=T))
```
