# Supplementary material for: Resources modify the associations between kin and fertility in historical Finland
Source: Evol Hum Sci. 2026 Jul 3;8:e30. doi: 10.1017/ehs.2026.10060 (PMC13435151; doi:10.1017/ehs.2026.10060)
Supplement: Supplementary file 1 [file S2513843X26100607sup001.zip › Supplementary information.docx]

Supplementary Table S1. Analysis of variance (ANOVA) for marriage. Significant p values are indicated in bold. χ2 = chi squared test statistic, DF = degrees of freedom, p_FDR_ = p value corrected for false discovery rate.

| **Variable** | **χ2** | **DF** | **p value** | **p_FDR_** |
| --- | --- | --- | --- | --- |
| region | 47.48 | 2 | **<0.0001** | **<0.0001** |
| sex | 16.29 | 1 | **0.0001** | **0.0005** |
| SES class | 4.67 | 1 | **0.0307** | 0.0894 |
| older brothers | 11.31 | 2 | **0.0035** | **0.0186** |
| older sisters | 7.09 | 2 | **0.0288** | 0.0865 |
| younger brothers | 0.16 | 2 | 0.9219 | 0.9649 |
| younger sisters | 1.77 | 2 | 0.4124 | 0.5631 |
| nephews | 6.10 | 1 | **0.0135** | 0.0542 |
| sex * SES class | 12.01 | 1 | **0.0005** | **0.0038** |
| sex * older brothers | 1.75 | 2 | 0.4164 | 0.5631 |
| SES class * older brothers | 4.91 | 2 | 0.0857 | 0.2006 |
| sex * older sisters | 1.56 | 2 | 0.4576 | 0.5937 |
| SES class * older sisters | 2.35 | 2 | 0.3088 | 0.4941 |
| sex * younger brothers | 7.55 | 2 | **0.0230** | 0.0761 |
| SES class * younger brothers | 0.71 | 2 | 0.7012 | 0.8310 |
| sex * younger sisters | 7.35 | 2 | **0.0254** | 0.0812 |
| SES class * younger sisters | 7.59 | 2 | **0.0225** | 0.0761 |
| sex * nephews | 0.06 | 1 | 0.8048 | 0.9106 |
| SES class * nephews | 0.71 | 1 | 0.3987 | 0.5547 |
| sex * SES class * older brothers | 0.03 | 2 | 0.9865 | 0.9865 |
| sex * SES class * older sisters | 17.86 | 2 | **0.0001** | **0.0012** |
| sex * SES class * younger brothers | 6.41 | 2 | **0.0405** | 0.1110 |
| sex * SES class * younger sisters | 2.99 | 2 | 0.2242 | 0.4221 |
| sex * SES class * nephews | 0.74 | 1 | 0.3900 | 0.5547 |

Supplementary Table S2. Pairwise comparisons of kin frequency classes compared to having no such kin alive in the binomial part (probability to remain unmarried) of the hurdle model of marriage. Significant p values are indicated in bold. OR = odds ratio, CL = confidence limits, t = t score, p_FDR_ = p value corrected for false discovery rate.

| **relative** | **group** | **comparison** | **OR** | **CL** | **t** | **p value** | **p_FDR_** |
| --- | --- | --- | --- | --- | --- | --- | --- |
| older brothers | landless men | 1 vs 0 | 1.048 | [0.690,1.592] | 0.22 | 0.8249 | 0.9106 |
|  |  | 2 or more vs 0 | 1.145 | [0.666,1.967] | 0.49 | 0.6246 | 0.7787 |
|  | landless women | 1 vs 0 | 1.091 | [0.757,1.573] | 0.47 | 0.6396 | 0.7872 |
|  |  | 2 or more vs 0 | 1.907 | [1.225,2.969] | 2.86 | **0.0042** | **0.0214** |
|  | landowner men | 1 vs 0 | 2.191 | [1.523,3.152] | 4.23 | **0.0000** | **0.0003** |
|  |  | 2 or more vs 0 | 2.792 | [1.804,4.320] | 4.61 | **0.0000** | **0.0001** |
|  | landowner women | 1 vs 0 | 1.301 | [0.906,1.869] | 1.43 | 0.1538 | 0.3142 |
|  |  | 2 or more vs 0 | 1.500 | [0.991,2.269] | 1.92 | 0.0550 | 0.1386 |
| older sisters | landless men | 1 vs 0 | 1.274 | [0.825,1.969] | 1.09 | 0.2745 | 0.4745 |
|  |  | 2 or more vs 0 | 1.384 | [0.809,2.368] | 1.19 | 0.2353 | 0.4344 |
|  | landless women | 1 vs 0 | 1.004 | [0.710,1.421] | 0.02 | 0.9809 | 0.9865 |
|  |  | 2 or more vs 0 | 1.223 | [0.782,1.911] | 0.88 | 0.3775 | 0.5547 |
|  | landowner men | 1 vs 0 | 1.348 | [0.912,1.990] | 1.50 | 0.1338 | 0.2793 |
|  |  | 2 or more vs 0 | 1.441 | [0.917,2.264] | 1.59 | 0.1130 | 0.2522 |
|  | landowner women | 1 vs 0 | 1.587 | [1.140,2.208] | 2.74 | **0.0062** | **0.0279** |
|  |  | 2 or more vs 0 | 1.437 | [0.944,2.189] | 1.69 | 0.0909 | 0.2077 |
| younger brothers | landless men | 1 vs 0 | 1.095 | [0.710,1.690] | 0.41 | 0.6802 | 0.8162 |
|  |  | 2 or more vs 0 | 1.086 | [0.683,1.727] | 0.35 | 0.7270 | 0.8511 |
|  | landless women | 1 vs 0 | 0.834 | [0.569,1.221] | -0.93 | 0.3511 | 0.5525 |
|  |  | 2 or more vs 0 | 0.841 | [0.569,1.242] | -0.87 | 0.3840 | 0.5547 |
|  | landowner men | 1 vs 0 | 0.981 | [0.655,1.468] | -0.09 | 0.9247 | 0.9649 |
|  |  | 2 or more vs 0 | 1.567 | [1.051,2.337] | 2.20 | **0.0277** | 0.0858 |
|  | landowner women | 1 vs 0 | 1.008 | [0.684,1.487] | 0.04 | 0.9676 | 0.9865 |
|  |  | 2 or more vs 0 | 1.239 | [0.842,1.825] | 1.09 | 0.2768 | 0.4745 |
| younger sisters | landless men | 1 vs 0 | 1.233 | [0.781,1.948] | 0.90 | 0.3691 | 0.5547 |
|  |  | 2 or more vs 0 | 1.313 | [0.817,2.112] | 1.13 | 0.2605 | 0.4630 |
|  | landless women | 1 vs 0 | 1.081 | [0.765,1.528] | 0.44 | 0.6592 | 0.8011 |
|  |  | 2 or more vs 0 | 0.836 | [0.563,1.243] | -0.88 | 0.3764 | 0.5547 |
|  | landowner men | 1 vs 0 | 1.121 | [0.738,1.704] | 0.54 | 0.5917 | 0.7474 |
|  |  | 2 or more vs 0 | 1.635 | [1.073,2.491] | 2.29 | **0.0221** | 0.0761 |
|  | landowner women | 1 vs 0 | 1.315 | [0.922,1.876] | 1.51 | 0.1306 | 0.2785 |
|  |  | 2 or more vs 0 | 1.229 | [0.836,1.808] | 1.05 | 0.2949 | 0.4941 |
| nephews | landless men | 1 or more vs 0 | 1.037 | [0.586,1.834] | 0.13 | 0.9005 | 0.9605 |
|  | landless women | 1 or more vs 0 | 0.807 | [0.491,1.325] | -0.85 | 0.3963 | 0.5547 |
|  | landowner men | 1 or more vs 0 | 0.861 | [0.544,1.362] | -0.64 | 0.5218 | 0.6680 |
|  | landowner women | 1 or more vs 0 | 0.736 | [0.476,1.137] | -1.38 | 0.1670 | 0.3340 |

Supplementary Table S3. Pairwise comparisons of kin frequency classes compared to having no such kin alive in the continuous part (age at first marriage) of the hurdle model of marriage. Significant p values are indicated in bold. est = difference in age at first marriage in years, CL = confidence limits, t = t score, p_FDR_ = p value corrected for false discovery rate.

| **relative** | **group** | **comparison** | **est** | **CL** | **t** | **p value** | **p_FDR_** |
| --- | --- | --- | --- | --- | --- | --- | --- |
| older brothers | landless men | 1 vs 0 | 0.553 | [0.156,0.951] | 2.73 | **0.0064** | **0.0279** |
|  |  | 2 or more vs 0 | 0.736 | [0.209,1.264] | 2.74 | **0.0062** | **0.0279** |
|  | landless women | 1 vs 0 | 0.212 | [-0.195,0.620] | 1.02 | 0.3069 | 0.4941 |
|  |  | 2 or more vs 0 | 0.382 | [-0.165,0.929] | 1.37 | 0.1714 | 0.3358 |
|  | landowner men | 1 vs 0 | 0.963 | [0.578,1.349] | 4.90 | **0.0000** | **0.0000** |
|  |  | 2 or more vs 0 | 1.489 | [1.010,1.967] | 6.10 | **0.0000** | **0.0000** |
|  | landowner women | 1 vs 0 | 0.608 | [0.222,0.994] | 3.09 | **0.0020** | **0.0115** |
|  |  | 2 or more vs 0 | 1.053 | [0.601,1.505] | 4.57 | **0.0000** | **0.0001** |
| older sisters | landless men | 1 vs 0 | 0.418 | [0.006,0.830] | 1.99 | **0.0468** | 0.1222 |
|  |  | 2 or more vs 0 | 0.646 | [0.109,1.183] | 2.36 | **0.0183** | 0.0703 |
|  | landless women | 1 vs 0 | 0.053 | [-0.346,0.452] | 0.26 | 0.7952 | 0.9106 |
|  |  | 2 or more vs 0 | 0.489 | [-0.047,1.025] | 1.79 | 0.0736 | 0.1767 |
|  | landowner men | 1 vs 0 | -0.012 | [-0.422,0.398] | -0.06 | 0.9546 | 0.9854 |
|  |  | 2 or more vs 0 | 0.281 | [-0.200,0.763] | 1.14 | 0.2523 | 0.4570 |
|  | landowner women | 1 vs 0 | 1.103 | [0.736,1.470] | 5.89 | **0.0000** | **0.0000** |
|  |  | 2 or more vs 0 | 1.752 | [1.296,2.208] | 7.53 | **0.0000** | **0.0000** |
| younger brothers | landless men | 1 vs 0 | -0.038 | [-0.454,0.378] | -0.18 | 0.8587 | 0.9262 |
|  |  | 2 or more vs 0 | 0.052 | [-0.382,0.487] | 0.24 | 0.8129 | 0.9106 |
|  | landless women | 1 vs 0 | 0.797 | [0.363,1.231] | 3.60 | **0.0003** | **0.0026** |
|  |  | 2 or more vs 0 | 0.529 | [0.085,0.972] | 2.34 | **0.0194** | 0.0717 |
|  | landowner men | 1 vs 0 | 0.213 | [-0.196,0.621] | 1.02 | 0.3074 | 0.4941 |
|  |  | 2 or more vs 0 | 0.171 | [-0.253,0.595] | 0.79 | 0.4291 | 0.5718 |
|  | landowner women | 1 vs 0 | 0.722 | [0.312,1.132] | 3.45 | **0.0006** | **0.0038** |
|  |  | 2 or more vs 0 | 1.387 | [0.975,1.799] | 6.60 | **0.0000** | **0.0000** |
| younger sisters | landless men | 1 vs 0 | -0.278 | [-0.709,0.154] | -1.26 | 0.2074 | 0.3981 |
|  |  | 2 or more vs 0 | -0.050 | [-0.498,0.397] | -0.22 | 0.8252 | 0.9106 |
|  | landless women | 1 vs 0 | 0.192 | [-0.222,0.607] | 0.91 | 0.3632 | 0.5547 |
|  |  | 2 or more vs 0 | -0.440 | [-0.874,-0.006] | -1.99 | **0.0471** | 0.1222 |
|  | landowner men | 1 vs 0 | 0.411 | [-0.011,0.834] | 1.91 | 0.0563 | 0.1386 |
|  |  | 2 or more vs 0 | 0.741 | [0.310,1.172] | 3.37 | **0.0008** | **0.0049** |
|  | landowner women | 1 vs 0 | 0.155 | [-0.234,0.543] | 0.78 | 0.4348 | 0.5718 |
|  |  | 2 or more vs 0 | -0.041 | [-0.446,0.365] | -0.20 | 0.8439 | 0.9206 |
| nephews | landless men | 1 or more vs 0 | -0.714 | [-1.281,-0.147] | -2.47 | **0.0135** | 0.0542 |
|  | landless women | 1 or more vs 0 | -0.614 | [-1.190,-0.037] | -2.09 | **0.0369** | 0.1042 |
|  | landowner men | 1 or more vs 0 | -0.391 | [-0.890,0.109] | -1.53 | 0.1251 | 0.2730 |
|  | landowner women | 1 or more vs 0 | -0.744 | [-1.205,-0.283] | -3.16 | **0.0016** | **0.0094** |

Supplementary Table S4. Analysis of variance (ANOVA) in the paternal kin model for lifetime number of children. Significant p values are indicated in bold. χ2 = chi squared test statistic, DF = degrees of freedom, p_FDR_ = p value corrected for false discovery rate.

| **Variable** | **χ2** | **DF** | **p value** | **p_FDR_** |
| --- | --- | --- | --- | --- |
| region | 43.58 | 2 | **<0.0001** | **<0.0001** |
| sex | 0.04 | 1 | 0.8429 | 0.9177 |
| SES class | 1.32 | 1 | 0.2508 | 0.5397 |
| paternal uncles | 6.36 | 2 | **0.0417** | 0.2866 |
| paternal cousins | 11.88 | 2 | **0.0026** | 0.0904 |
| younger brothers | 6.50 | 2 | **0.0389** | 0.2866 |
| younger sisters | 4.25 | 2 | 0.1193 | 0.4238 |
| sex * SES class | 0.40 | 1 | 0.5259 | 0.7183 |
| sex * paternal uncles | 8.70 | 2 | **0.0129** | 0.1832 |
| SES class * paternal uncles | 1.28 | 2 | 0.5286 | 0.7183 |
| sex * paternal cousins | 9.31 | 2 | **0.0095** | 0.1832 |
| SES class * paternal cousins | 3.17 | 2 | 0.2051 | 0.4832 |
| sex * younger brothers | 5.42 | 2 | 0.0665 | 0.3459 |
| SES class * younger brothers | 2.31 | 2 | 0.3151 | 0.5955 |
| sex * younger sisters | 0.12 | 2 | 0.9429 | 0.9653 |
| SES class * younger sisters | 0.24 | 2 | 0.8874 | 0.9364 |
| sex * SES class * paternal uncles | 2.19 | 2 | 0.3349 | 0.6087 |
| sex * SES class * paternal cousins | 3.25 | 2 | 0.1971 | 0.4802 |
| sex * SES class * younger brothers | 5.05 | 2 | 0.0800 | 0.3721 |
| sex * SES class * younger sisters | 0.98 | 2 | 0.6124 | 0.7782 |

Supplementary Table S5. Pairwise comparisons of kin frequency classes compared to having no such kin alive in the binomial part (probability of lifetime childlessness) of the paternal kin hurdle model of children. Significant p values are indicated in bold. OR = odds ratio, CL = confidence limits, z = z score, p_FDR_ = p value corrected for false discovery rate.

| **relative** | **group** | **comparison** | **OR** | **CL** | **z** | **p value** | **p_FDR_** |
| --- | --- | --- | --- | --- | --- | --- | --- |
| paternal uncles | landless men | 1 vs 0 | 0.721 | [0.347,1.498] | -0.88 | 0.3804 | 0.6262 |
|  |  | 2 or more vs 0 | 0.551 | [0.251,1.208] | -1.49 | 0.1368 | 0.4328 |
|  | landless women | 1 vs 0 | 2.145 | [0.741,6.208] | 1.41 | 0.1591 | 0.4685 |
|  |  | 2 or more vs 0 | 2.414 | [0.800,7.282] | 1.56 | 0.1177 | 0.4238 |
|  | landowner men | 1 vs 0 | 0.776 | [0.388,1.553] | -0.72 | 0.4739 | 0.6908 |
|  |  | 2 or more vs 0 | 1.919 | [0.946,3.892] | 1.81 | 0.0710 | 0.3459 |
|  | landowner women | 1 vs 0 | 1.307 | [0.693,2.464] | 0.83 | 0.4087 | 0.6509 |
|  |  | 2 or more vs 0 | 0.639 | [0.287,1.422] | -1.10 | 0.2729 | 0.5635 |
| paternal cousins | landless men | 1 vs 0 | 1.391 | [0.624,3.102] | 0.81 | 0.4203 | 0.6620 |
|  |  | 2 or more vs 0 | 0.900 | [0.379,2.138] | -0.24 | 0.8119 | 0.9009 |
|  | landless women | 1 vs 0 | 0.452 | [0.146,1.394] | -1.38 | 0.1669 | 0.4685 |
|  |  | 2 or more vs 0 | 0.226 | [0.071,0.721] | -2.51 | **0.0120** | 0.1832 |
|  | landowner men | 1 vs 0 | 0.733 | [0.382,1.406] | -0.94 | 0.3495 | 0.6176 |
|  |  | 2 or more vs 0 | 0.368 | [0.178,0.761] | -2.70 | **0.0070** | 0.1717 |
|  | landowner women | 1 vs 0 | 0.813 | [0.390,1.693] | -0.55 | 0.5799 | 0.7500 |
|  |  | 2 or more vs 0 | 0.659 | [0.311,1.394] | -1.09 | 0.2752 | 0.5635 |
| younger brothers | landless men | 1 vs 0 | 1.079 | [0.551,2.112] | 0.22 | 0.8254 | 0.9100 |
|  |  | 2 or more vs 0 | 0.615 | [0.288,1.313] | -1.26 | 0.2093 | 0.4864 |
|  | landless women | 1 vs 0 | 2.215 | [0.853,5.751] | 1.63 | 0.1022 | 0.4132 |
|  |  | 2 or more vs 0 | 1.586 | [0.576,4.371] | 0.89 | 0.3721 | 0.6262 |
|  | landowner men | 1 vs 0 | 0.896 | [0.486,1.653] | -0.35 | 0.7251 | 0.8518 |
|  |  | 2 or more vs 0 | 1.112 | [0.611,2.024] | 0.35 | 0.7280 | 0.8518 |
|  | landowner women | 1 vs 0 | 0.649 | [0.336,1.253] | -1.29 | 0.1977 | 0.4802 |
|  |  | 2 or more vs 0 | 0.714 | [0.379,1.346] | -1.04 | 0.2981 | 0.5886 |
| younger sisters | landless men | 1 vs 0 | 0.706 | [0.343,1.455] | -0.94 | 0.3452 | 0.6176 |
|  |  | 2 or more vs 0 | 0.857 | [0.414,1.775] | -0.42 | 0.6779 | 0.8154 |
|  | landless women | 1 vs 0 | 0.282 | [0.096,0.825] | -2.31 | **0.0208** | 0.2217 |
|  |  | 2 or more vs 0 | 0.613 | [0.248,1.516] | -1.06 | 0.2897 | 0.5863 |
|  | landowner men | 1 vs 0 | 0.694 | [0.385,1.250] | -1.22 | 0.2239 | 0.5066 |
|  |  | 2 or more vs 0 | 0.532 | [0.285,0.992] | -1.99 | 0.0471 | 0.2877 |
|  | landowner women | 1 vs 0 | 0.616 | [0.312,1.215] | -1.40 | 0.1617 | 0.4685 |
|  |  | 2 or more vs 0 | 1.152 | [0.620,2.140] | 0.45 | 0.6542 | 0.8153 |

Supplementary Table S6. Pairwise comparisons of kin frequency classes compared to having no such kin alive in the discrete part (lifetime number of children) of the paternal kin hurdle model of children. Significant p values are indicated in bold. % diff = percent difference in mean lifetime number of children, CL = confidence limits, t = t score, p_FDR_ = p value corrected for false discovery rate.

| **relative** | **group** | **comparison** | **% diff** | **CL** | **t** | **p value** | **p_FDR_** |
| --- | --- | --- | --- | --- | --- | --- | --- |
| paternal uncles | landless men | 1 vs 0 | 7.05 | [-3.50,18.75] | 1.29 | 0.1981 | 0.4802 |
|  |  | 2 or more vs 0 | -5.97 | [-15.72,4.91] | -1.10 | 0.2704 | 0.5635 |
|  | landless women | 1 vs 0 | 7.25 | [-2.85,18.40] | 1.39 | 0.1653 | 0.4685 |
|  |  | 2 or more vs 0 | 12.30 | [1.40,24.37] | 2.23 | **0.0259** | 0.2345 |
|  | landowner men | 1 vs 0 | 7.63 | [-0.46,16.39] | 1.84 | 0.0653 | 0.3459 |
|  |  | 2 or more vs 0 | 0.87 | [-7.44,9.93] | 0.20 | 0.8430 | 0.9177 |
|  | landowner women | 1 vs 0 | 2.32 | [-5.36,10.61] | 0.58 | 0.5648 | 0.7473 |
|  |  | 2 or more vs 0 | 5.45 | [-2.85,14.45] | 1.27 | 0.2046 | 0.4832 |
| paternal cousins | landless men | 1 vs 0 | -5.87 | [-17.13,6.92] | -0.93 | 0.3519 | 0.6176 |
|  |  | 2 or more vs 0 | 10.65 | [-2.79,25.94] | 1.53 | 0.1257 | 0.4238 |
|  | landless women | 1 vs 0 | -2.76 | [-14.37,10.44] | -0.43 | 0.6667 | 0.8154 |
|  |  | 2 or more vs 0 | -4.80 | [-16.11,8.04] | -0.76 | 0.4464 | 0.6776 |
|  | landowner men | 1 vs 0 | -0.73 | [-9.43,8.81] | -0.16 | 0.8763 | 0.9362 |
|  |  | 2 or more vs 0 | 4.82 | [-4.38,14.91] | 1.01 | 0.3150 | 0.5955 |
|  | landowner women | 1 vs 0 | -4.43 | [-12.85,4.81] | -0.96 | 0.3362 | 0.6087 |
|  |  | 2 or more vs 0 | -3.00 | [-11.36,6.14] | -0.66 | 0.5072 | 0.7183 |
| younger brothers | landless men | 1 vs 0 | 7.87 | [-1.91,18.61] | 1.56 | 0.1181 | 0.4238 |
|  |  | 2 or more vs 0 | 12.91 | [2.75,24.07] | 2.53 | **0.0116** | 0.1832 |
|  | landless women | 1 vs 0 | -7.73 | [-15.84,1.17] | -1.71 | 0.0870 | 0.3837 |
|  |  | 2 or more vs 0 | 4.20 | [-4.56,13.76] | 0.92 | 0.3590 | 0.6237 |
|  | landowner men | 1 vs 0 | -0.96 | [-7.66,6.24] | -0.27 | 0.7879 | 0.8857 |
|  |  | 2 or more vs 0 | 11.30 | [3.65,19.51] | 2.95 | **0.0032** | 0.0917 |
|  | landowner women | 1 vs 0 | -0.18 | [-7.13,7.29] | -0.05 | 0.9605 | 0.9776 |
|  |  | 2 or more vs 0 | 2.42 | [-4.68,10.05] | 0.65 | 0.5146 | 0.7183 |
| younger sisters | landless men | 1 vs 0 | 7.61 | [-2.34,18.58] | 1.48 | 0.1384 | 0.4328 |
|  |  | 2 or more vs 0 | 10.33 | [0.07,21.65] | 1.97 | **0.0485** | 0.2877 |
|  | landless women | 1 vs 0 | 9.95 | [1.06,19.61] | 2.21 | **0.0274** | 0.2356 |
|  |  | 2 or more vs 0 | 10.88 | [1.44,21.21] | 2.27 | **0.0230** | 0.2217 |
|  | landowner men | 1 vs 0 | 5.96 | [-1.46,13.94] | 1.56 | 0.1181 | 0.4238 |
|  |  | 2 or more vs 0 | 7.04 | [-0.47,15.1] | 1.83 | 0.0667 | 0.3459 |
|  | landowner women | 1 vs 0 | 6.10 | [-0.99,13.7] | 1.68 | 0.0932 | 0.4008 |
|  |  | 2 or more vs 0 | 14.10 | [6.42,22.33] | 3.71 | **0.0002** | **0.0086** |

Supplementary Table S7. Analysis of variance (ANOVA) in the maternal kin model for lifetime number of children. Significant p values are indicated in bold. χ2 = chi squared test statistic, DF = degrees of freedom, p_FDR_ = p value corrected for false discovery rate.

| **Variable** | **χ2** | **DF** | **p value** | **p_FDR_** |
| --- | --- | --- | --- | --- |
| region | 35.00 | 2 | **<0.0001** | **<0.0001** |
| sex | 0.17 | 1 | 0.6779 | 0.8154 |
| SES class | 0.14 | 1 | 0.7130 | 0.8458 |
| maternal grandmother | 0.75 | 1 | 0.3859 | 0.6262 |
| maternal cousins | 3.37 | 2 | 0.1856 | 0.4802 |
| younger brothers | 6.07 | 2 | **0.0481** | 0.2877 |
| younger sisters | 1.86 | 2 | 0.3939 | 0.6331 |
| nieces | 0.76 | 1 | 0.3823 | 0.6262 |
| sex * SES class | 2.05 | 1 | 0.1518 | 0.4661 |
| sex * maternal grandmother | 0.01 | 1 | 0.9410 | 0.9653 |
| SES class * maternal grandmother | 2.35 | 1 | 0.1257 | 0.4238 |
| sex * maternal cousins | 5.31 | 2 | 0.0705 | 0.3459 |
| SES class * maternal cousins | 0.05 | 2 | 0.9758 | 0.9845 |
| sex * younger brothers | 2.28 | 2 | 0.3201 | 0.5985 |
| SES class * younger brothers | 2.34 | 2 | 0.3109 | 0.5955 |
| sex * younger sisters | 3.70 | 2 | 0.1574 | 0.4685 |
| SES class * younger sisters | 2.67 | 2 | 0.2632 | 0.5589 |
| sex * nieces | 2.30 | 1 | 0.1293 | 0.4238 |
| SES class * nieces | 0.02 | 1 | 0.8867 | 0.9364 |
| sex * SES class * maternal grandmother | 0.41 | 1 | 0.5206 | 0.7183 |
| sex * SES class * maternal cousins | 1.44 | 2 | 0.4857 | 0.6961 |
| sex * SES class * younger brothers | 1.63 | 2 | 0.4419 | 0.6776 |
| sex * SES class * younger sisters | 3.25 | 2 | 0.1971 | 0.4802 |
| sex * SES class * nieces | 0.50 | 1 | 0.4810 | 0.6952 |

Supplementary Table S8. Pairwise comparisons of kin frequency classes compared to having no such kin alive in the binomial part (probability of lifetime childlessness) of the maternal kin hurdle model of children. Significant p values are indicated in bold. OR = odds ratio, CL = confidence limits, z = z score, p_FDR_ = p value corrected for false discovery rate.

| **relative** | **group** | **comparison** | **OR** | **CL** | **z** | **p value** | **p_FDR_** |
| --- | --- | --- | --- | --- | --- | --- | --- |
| maternal grandmother | landless men | alive vs dead | 1.234 | [0.615,2.478] | 0.59 | 0.5544 | 0.7392 |
|  | landless women | alive vs dead | 1.433 | [0.725,2.831] | 1.04 | 0.3003 | 0.5886 |
|  | landowner men | alive vs dead | 1.087 | [0.599,1.970] | 0.27 | 0.7845 | 0.8857 |
|  | landowner women | alive vs dead | 0.817 | [0.441,1.515] | -0.64 | 0.5214 | 0.7183 |
| maternal cousins | landless men | 1 vs 0 | 0.884 | [0.341,2.293] | -0.25 | 0.8003 | 0.8939 |
|  |  | 2 or more vs 0 | 1.037 | [0.418,2.569] | 0.08 | 0.9380 | 0.9653 |
|  | landless women | 1 vs 0 | 0.527 | [0.212,1.312] | -1.38 | 0.1689 | 0.4685 |
|  |  | 2 or more vs 0 | 0.794 | [0.358,1.760] | -0.57 | 0.5699 | 0.7482 |
|  | landowner men | 1 vs 0 | 0.384 | [0.171,0.860] | -2.33 | **0.0200** | 0.2217 |
|  |  | 2 or more vs 0 | 0.732 | [0.405,1.323] | -1.03 | 0.3012 | 0.5886 |
|  | landowner women | 1 vs 0 | 1.065 | [0.530,2.140] | 0.18 | 0.8596 | 0.9299 |
|  |  | 2 or more vs 0 | 0.671 | [0.347,1.298] | -1.18 | 0.2362 | 0.5208 |
| younger brothers | landless men | 1 vs 0 | 1.204 | [0.584,2.482] | 0.50 | 0.6154 | 0.7782 |
|  |  | 2 or more vs 0 | 1.006 | [0.451,2.243] | 0.01 | 0.9885 | 0.9885 |
|  | landless women | 1 vs 0 | 1.064 | [0.513,2.205] | 0.17 | 0.8682 | 0.9333 |
|  |  | 2 or more vs 0 | 0.842 | [0.383,1.851] | -0.43 | 0.6688 | 0.8154 |
|  | landowner men | 1 vs 0 | 0.508 | [0.271,0.949] | -2.12 | **0.0337** | 0.2763 |
|  |  | 2 or more vs 0 | 0.474 | [0.249,0.903] | -2.27 | **0.0232** | 0.2217 |
|  | landowner women | 1 vs 0 | 0.641 | [0.336,1.223] | -1.35 | 0.1772 | 0.4802 |
|  |  | 2 or more vs 0 | 0.771 | [0.408,1.457] | -0.80 | 0.4234 | 0.6620 |
| younger sisters | landless men | 1 vs 0 | 0.859 | [0.399,1.850] | -0.39 | 0.6977 | 0.8334 |
|  |  | 2 or more vs 0 | 0.957 | [0.441,2.077] | -0.11 | 0.9105 | 0.9549 |
|  | landless women | 1 vs 0 | 1.124 | [0.560,2.254] | 0.33 | 0.7430 | 0.8577 |
|  |  | 2 or more vs 0 | 0.739 | [0.331,1.647] | -0.74 | 0.4595 | 0.6798 |
|  | landowner men | 1 vs 0 | 0.435 | [0.224,0.844] | -2.46 | **0.0138** | 0.1832 |
|  |  | 2 or more vs 0 | 0.662 | [0.355,1.234] | -1.30 | 0.1942 | 0.4802 |
|  | landowner women | 1 vs 0 | 0.966 | [0.504,1.852] | -0.10 | 0.9182 | 0.9572 |
|  |  | 2 or more vs 0 | 1.359 | [0.735,2.514] | 0.98 | 0.3278 | 0.6062 |
| nieces | landless men | 1 or more vs 0 | 0.446 | [0.131,1.525] | -1.29 | 0.1982 | 0.4802 |
|  | landless women | 1 or more vs 0 | 0.722 | [0.261,1.995] | -0.63 | 0.5304 | 0.7183 |
|  | landowner men | 1 or more vs 0 | 0.824 | [0.418,1.622] | -0.56 | 0.5748 | 0.7490 |
|  | landowner women | 1 or more vs 0 | 0.768 | [0.385,1.530] | -0.75 | 0.4530 | 0.6776 |

Supplementary Table S9. Pairwise comparisons of kin frequency classes compared to having no such kin alive in the discrete part (lifetime number of children) of the maternal kin hurdle model of children. Significant p values are indicated in bold. % diff = percent difference in mean lifetime number of children, CL = confidence limits, t = t score, p_FDR_ = p value corrected for false discovery rate.

| **relative** | **group** | **comparison** | **% diff** | **CL** | **t** | **p value** | **p_FDR_** |
| --- | --- | --- | --- | --- | --- | --- | --- |
| maternal grandmother | landless men | alive vs dead | 3.91 | [-4.71,13.31] | 0.87 | 0.3859 | 0.6262 |
|  | landless women | alive vs dead | 3.45 | [-4.90,12.53] | 0.79 | 0.4298 | 0.6660 |
|  | landowner men | alive vs dead | 13.12 | [5.90,20.84] | 3.66 | **0.0002** | **0.0086** |
|  | landowner women | alive vs dead | 7.31 | [0.29,14.82] | 2.04 | **0.0410** | 0.2866 |
| maternal cousins | landless men | 1 vs 0 | 7.44 | [-4.95,21.46] | 1.15 | 0.2510 | 0.5397 |
|  |  | 2 or more vs 0 | 11.12 | [-0.95,24.66] | 1.80 | 0.0724 | 0.3459 |
|  | landless women | 1 vs 0 | -11.35 | [-21.21,-0.25] | -2.00 | **0.0453** | 0.2877 |
|  |  | 2 or more vs 0 | -2.62 | [-12.63,8.53] | -0.48 | 0.6314 | 0.7927 |
|  | landowner men | 1 vs 0 | 8.56 | [-1.12,19.18] | 1.72 | 0.0847 | 0.3834 |
|  |  | 2 or more vs 0 | 10.90 | [2.27,20.26] | 2.50 | **0.0123** | 0.1832 |
|  | landowner women | 1 vs 0 | 0.12 | [-8.59,9.66] | 0.03 | 0.9788 | 0.9845 |
|  |  | 2 or more vs 0 | 8.34 | [-0.19,17.61] | 1.91 | 0.0556 | 0.3188 |
| younger brothers | landless men | 1 vs 0 | 6.76 | [-1.90,16.17] | 1.52 | 0.1296 | 0.4238 |
|  |  | 2 or more vs 0 | 11.95 | [2.17,22.66] | 2.42 | **0.0155** | 0.1904 |
|  | landless women | 1 vs 0 | -2.78 | [-11.19,6.43] | -0.61 | 0.5411 | 0.7271 |
|  |  | 2 or more vs 0 | 5.69 | [-3.53,15.80] | 1.19 | 0.2349 | 0.5208 |
|  | landowner men | 1 vs 0 | -1.95 | [-8.65,5.24] | -0.55 | 0.5852 | 0.7512 |
|  |  | 2 or more vs 0 | 6.08 | [-1.19,13.89] | 1.63 | 0.1033 | 0.4132 |
|  | landowner women | 1 vs 0 | -1.07 | [-8.02,6.41] | -0.29 | 0.7733 | 0.8808 |
|  |  | 2 or more vs 0 | 6.53 | [-1.16,14.82] | 1.65 | 0.0982 | 0.4120 |
| younger sisters | landless men | 1 vs 0 | -5.87 | [-13.83,2.82] | -1.34 | 0.1794 | 0.4802 |
|  |  | 2 or more vs 0 | -1.97 | [-10.72,7.65] | -0.42 | 0.6772 | 0.8154 |
|  | landless women | 1 vs 0 | 3.22 | [-5.14,12.31] | 0.74 | 0.4624 | 0.6798 |
|  |  | 2 or more vs 0 | 9.78 | [0.48,19.94] | 2.07 | **0.0389** | 0.2866 |
|  | landowner men | 1 vs 0 | 2.81 | [-4.32,10.47] | 0.76 | 0.4503 | 0.6776 |
|  |  | 2 or more vs 0 | 5.90 | [-1.64,14.01] | 1.52 | 0.1284 | 0.4238 |
|  | landowner women | 1 vs 0 | -1.19 | [-7.87,5.98] | -0.34 | 0.7377 | 0.8573 |
|  |  | 2 or more vs 0 | 5.54 | [-1.59,13.18] | 1.51 | 0.1306 | 0.4238 |
| nieces | landless men | 1 or more vs 0 | -4.58 | [-14.09,6.00] | -0.87 | 0.3823 | 0.6262 |
|  | landless women | 1 or more vs 0 | 6.97 | [-3.90,19.07] | 1.23 | 0.2178 | 0.4995 |
|  | landowner men | 1 or more vs 0 | -3.65 | [-11.22,4.57] | -0.89 | 0.3732 | 0.6262 |
|  | landowner women | 1 or more vs 0 | 1.17 | [-5.98,8.86] | 0.31 | 0.7566 | 0.8676 |
